# In the context of the triple burden of malnutrition: A systematic review of gene-diet interactions and nutritional status

**DOI:** 10.1080/10408398.2022.2131727

**Published:** 2022-10-12

**Authors:** Pui Yee Tan, J. Bernadette Moore, Ling Bai, GuYuan Tang, Yun Yun Gong

**Affiliations:** a School of Food Science and Nutrition, Faculty of Environment, University of Leeds, Leeds, United Kingdom; b School of Psychology, University of East Anglia, Norwich, United Kingdom

**Keywords:** Malnutrition, dietary pattern, gene-diet interaction, genetic risk scores, SNPs, tripe burden of malnutrition, obesity

## Abstract

Genetic background interacts with dietary components to modulate nutritional health status. This study aimed to review the evidence for gene-diet interactions in all forms of malnutrition. A comprehensive systematic literature search was conducted through April 2021 to identify observational and intervention studies reporting the effects of gene-diet interactions in over-nutrition, under-nutrition and micronutrient status. Risk of publication bias was assessed using the Quality Criteria Checklist and a tool specifically designed for gene-diet interaction research. 167 studies from 27 populations were included. The majority of studies investigated single nucleotide polymorphisms (SNPs) in overnutrition (n = 158). Diets rich in whole grains, vegetables, fruits and low in total and saturated fats, such as Mediterranean and DASH diets, showed promising effects for reducing obesity risk among individuals who had higher genetic risk scores for obesity, particularly the risk alleles carriers of *FTO* rs9939609, rs1121980 and rs1421085. Other SNPs in *MC4R*, *PPARG* and *APOA5* genes were also commonly studied for interaction with diet on overnutrition though findings were inconclusive. Only limited data were found related to undernutrition (n = 1) and micronutrient status (n = 9). The findings on gene-diet interactions in this review highlight the importance of personalized nutrition, and more research on undernutrition and micronutrient status is warranted.

## Introduction

Malnutrition in all its forms, including micronutrient deficiencies, undernutrition and overnutrition, remains a leading cause of global mortality and morbidity (Popkin, Corvalan, and Grummer-Strawn 2020). Suboptimal diets are the critical environmental factor involved in the development of malnutrition and diet-related disease (Afshin et al. 2019). However, individual dietary requirements differ from person to person, with genetic variation influencing individual’s dietary requirements and nutritional status (Stover 2006). Thus, nutritional requirements and dietary recommendations may not always be generalizable to entire populations. In addition, dietary components can modulate the expression of genes involved in metabolic pathways determining nutritional status and health outcomes (Fenech et al. 2011). This can be mediated either through nutrient-regulated transcription factors, or through epigenetic mechanisms such as DNA methylation and histone modification (Tiffon 2018).

Current government recommended nutrient intakes (e.g., recommended dietary allowances (RDAs) or safe upper limits) are designed for the general population based on different metabolic outcomes or population subgroups, such as the elderly, pregnant or lactating women (Ordovas et al. 2018). These recommendations are not optimized for genetic subgroups that may differ in response to different dietary components. The understanding that there is no one-size-fits-all diet contributed to the emergence of nutritional genomics research. This includes both nutrigenetics, which studies the role of genetic variations on nutrient metabolism and diet-related disease, and nutrigenomics, which examines the role of nutrients or dietary patterns on gene expression (transcriptomics); along with more broadly, proteomics and metabolomics (Phillips 2013). Requisite to both nutrigenomics and nutrigenetics research, is an understanding of nutrition, genetics, biochemistry and application of a range of ‘omics’ technologies to investigate the complex interaction between genetic and environmental factors relevant to metabolic health and disease (Matusheski et al. 2021). Precision nutrition is a more recent term, often used interchangeably with personalized nutrition, nutritional genomics, nutrigenomics or nutrigenetics (Bush et al. 2020). The objective of precision nutrition is the identification of personalized nutritional recommendations that are tailored according to an individual’s biological requirements and predicted response to dietary intervention based on genetic, metabolomic and microbiome profiling (de Toro-Martin et al. 2017). Research in this area aims to provide a better understanding of nutrient-gene interactions with the ultimate goal of developing personalized nutrition strategies for optimal health and disease prevention.

A growing number of companies have offered the services of direct-to-consumer genetic testing (DTC-GT) personalized dietary advice in the past decade (Phillips 2016). Next-generation sequencing (NGS) technologies that facilitate the sequencing of whole genome at an unprecedented speed and simultaneous high-throughput testing of multiple genes (Metzker 2010), have further enabled nutrigenomics and nutrigenetics research. The advances in sequencing technology have driven down the cost of human sequencing, with a current estimated sequencing cost of $1,000 US dollars per genome (Moore 2020). These advantages have facilitated and accelerated research and development of precision nutrition strategies for the prevention and treatment for nutritional conditions. However, questions remain about efficacy, cost-benefit and accessibility, with the current scientific evidence suggesting that it is still premature to apply the use of precision nutrition in the community (Moore 2020).

The most commonly investigated type of genetic variation in genome-wide association studies (GWAS) has been single base pair differences, termed single nucleotide polymorphisms (SNPs) (Bush and Moore 2012). Other types of genetic variation include structural variants (SVs), such as insertions and deletions of short DNA fragments (INDELs), and copy number variants (CNV) where the number of copies of particular genes varies between individuals (Feuk, Carson, and Scherer 2006). To date, most of the nutritional genomics research has focused on SNPs, therefore these were the central focus for this review. Moreover, in contrast to obesity, far fewer studies have investigated genetic variants associated with under-nutrition or micronutrient status, and not all micronutrients have been studied. Therefore, this study aimed to systematically review the current evidence on the effects of gene-diet interactions on nutritional status including undernutrition (stunting, wasting and underweight), overnutrition (overweight and obesity) and micronutrient status; particularly that of iron, zinc, folate and vitamin A. This included examining the effects of genetic variants on phenotypes in response to nutrient or diet.

## Methods

This systematic review was conducted by three independent reviewers following PRISMA guidelines and was prospectively registered at PROSPERO (registration no. CRD42021245115).

### Eligibility criteria

The PICOS criteria for inclusion and exclusion in this review are shown in Table 1. Both observational studies and intervention trials involving human participants of all age, gender and ethnicity that investigated the effect of gene-diet interactions defined as the combined effect of the two exposures on nutritional status were included.

**Table 1.** PICOS criteria for inclusion and exclusion of studies.

Parameter	Inclusion criteria	Exclusion criteria
**Population**	Human participants <65 years old of all gender or ethnicity.	Animal studies; human participants who were pregnant, using drugs or treatments in past 12 months, suffering from infectious diseases, or previously diagnosed conditions that may lead to changes in metabolic and nutritional needs including postoperative recovery, all types of cancer, renal disorders, mental health disorders, and endocrine disorders.
**Exposure or Intervention**	Studies that investigated gene-diet interactions, which defined as the combined effects of the two exposures including genetic (e.g., single nucleotide polymorphisms, gene variation and mutation, gene expression and epigenetics such as microRNA expression, DNA methylation, acetylation, and histone modification) and dietary factors on nutritional status.	Studies that reported individual exposure effect.
**Comparison**	Those who did not expose to the two exposures.	
**Outcome**	Malnutrition including undernutrition (stunting, wasting, underweight, and micronutrient deficiencies) and overnutrition (overweight and obesity). The parameters included height, weight status and adiposity such as body weight, body mass index (BMI), height-for-age, weight-for-age, weight-for-height, body fat and waist circumference. Micronutrient deficiencies including iron, zinc, vitamin A, and folate which assessed using blood, serum, or plasma samples.	Studies not reporting primary outcomes of interest.
**Study design**	Observational studies including cross-sectional, case-control and cohort studies and interventional studies including randomized and non-randomized controlled trials	Letters or case reports

### Search strategy

Four databases, PubMed, Scopus, Embase (Ovid) and Web of Science, were systematically searched using the combination of keywords and terms (e.g., MeSH and Emtree) through 30^th^ April 2021. The search was conducted using the following keywords from three main themes: i) gene exposure (“single nucleotide polymorphism*” OR SNP* OR gene* OR genetic* OR polymorphism* OR genotype* OR allele* OR variant* OR mutant* OR expression* OR miRNA* OR epigenetic* OR methylation* OR acetylation* OR “histone modification*”) AND ii) dietary exposure (diet* OR intake* OR pattern* OR consumption* OR eating OR meal), AND iii) nutritional status including anthropometric indicators for under and over nutrition (weight OR adiposity OR “body mass index” OR BMI OR overweight OR obes* OR underweight OR stunt* OR wasting OR underweight OR “height-for-age” OR “weight-for-height” OR “weight-for-age”) and micronutrient status ((iron OR ferritin OR transferrin OR h?emoglobin OR folate OR folic acid OR “Vitamin B9” OR retinol OR “vitamin A” OR zinc) AND (blood OR plasma OR serum) AND (deficien* OR insufficien* OR inadequa*)). The details of the search strategies developed for each database are documented Table S1.

### Study selection

Screening of the identified studies and selection of studies for inclusion in this review based on the eligibility criteria detailed above were performed independently by three reviewers using both Endnote (Endnote X7.7.1, Thomson Reuters 2016) and Rayyan (http://rayyan.qcri.org) tools. The final decision regarding the eligibility of articles was made by agreement between the three reviewers. Disagreement between reviewers was resolved by discussion and by other reviewers when necessary.

### Data extraction

A standardized data extraction form was utilized to obtain the following information: author, year of publication, study design, year of study, sample size, country or population, sample characteristics (e.g., gender, age and BMI), exposure or intervention (both gene and dietary exposures), outcome measures (e.g., indicators related to malnutrition), main findings (e.g., β coefficient, odds ratio, differences in mean) and statistical analysis. In the case of missing data or unclear pieces of information, it was considered that the authors did not report such variables.

### Risk of bias assessment

Risk of bias in the individual studies included was assessed by independent reviewers using the Academy of Nutrition and Dietetics, Quality Criteria Checklist (2016 Evidence Analysis Manual, Academy of Nutrition and Dietetics). The 10 questions of the checklist focus on: (1) how clear the research question was, (2) selection of participants, (3) randomization/group comparability, (4) description of withdrawals, (5) how the blinding was conducted, (6) whether study procedures were described clearly, (7) whether the outcomes were clearly defined and the validity of the measurements, (8) were appropriate statistical analyses applied, (9) did the results support author’s conclusions, and (10) was there funding or sponsorship bias. To be rated low risk of bias, each of criteria 2, 3, 6 and 7 must be met and the majority of 10 criteria overall. Any of criteria 2, 3, 6 and 7 not being met resulted in a neutral rating. If most criteria are not met, the article was rated high risk of bias.

In addition, a specific assessment to evaluate the methodological quality of gene-diet interaction research was performed following the criteria important for genetic association studies (Campbell and Rudan 2002; Dietrich et al. 2019). The score was based on eight items (Table S2): (1) interaction as primary study goal, (2) statistical test for interaction, (3) correction for multiple testing, (4) correction for ethnicity or population structure, (5) Hardy‐Weinberg equilibrium testing, (6) test for group similarity at baseline or the comparability of case and control, (7) sample size or power analysis, and (8) sufficient details of study procedure. Based on a scoring of positive (+1), neutral (0) or negative (-1) for each item, the total points for each paper could range from −8 to 8; and were assessed as: high (6 to 8 points), neutral quality (2 to 5 points), and low (−8 to 1 points) quality.

**Table 2.** Summary of observational studies (n = 101) examining diet-gene interactions and weight status.

Dietary components	Reference	Name of studies	Population	Sample size (female %; age in years; BMI in kg/m^2^)	SNPs (alleles) or GRS	Impacts on outcome measures^a^	Risk of bias (general)^1^	Risk of bias (genetic)^2^
Alcohol	Latella et al. 2009	IMMIDIET	Belgium, Italy and England	974 (50%; 25-74y; NS)	*ADH1C* rs698 (A > G)	↑	Low	Low
	Rohde et al. 2017	MONICA, DCH and INTER99	Denmark	7,208 (50%; 41y; 29 kg/m^2^)	Unweighted GRS: 50 SNPs; *DNM3-PIGC* rs1011731 (T > C); *TRA2B* rs7647305 (T > C); *TNNI3K* rs1514175 (C > T)	↓	Low	Low
	Wang et al. 2016		China	2,958 (54%; 52 ± 7y; 24.4 ± 3.4 kg/m^2^)	*ALDH2* rs671 (A > G)	↓	Low	Low
	Young, Wauthier, and Donnelly 2016	UK biobank	UK	119,132 (48%; 40-69y; 27.4 ± 4.8 kg/m^2^)	*FTO* rs1421085 (T > C)	↓	Low	Low
AHEI-2010, AMED and DASH scores	Ding et al. 2018	NHS, HPFS and WGHS	US and Europe	31,058 (89%; 30-75y; NS)	Unweighted GRS: 97 SNPs	↓	Low	Low
	Wang et al. 2018	NHS and HPFS	US	14,046 (63%; 30-75y; NS)	Weighted (β) GRS: 77 SNPs	↓	Low	Neutral
Breastfeeding	Dedoussis et al. 2011	GENDAI, GENESIS, ALSPAC	Greece and UK	7,837 (NS; 1-12y; NS)	*FTO* rs9939609 (T > A)	↓	Low	Low
	Mook-Kanamori et al. 2009	Generation R	Europe	3,432 (NS; 1.5-18m; NS)	*PPARG* rs1801282 (C > G)	↓	Low	Neutral
	Wu, Lye, and Briollais 2017	ALSPAC	UK	5,590 (49%; 0-16y; NS)	*FTO* rs9939609 (T > A)	↓	Low	Low
Calcium	Larsen et al. 2014b	MONICA, DCH and Inter99	Denmark	7,659 (49-52%; 31-61y; NS)	Unweighted GRS: 54 SNPs	↓	Low	Low
	Marcos-Pasero et al. 2019	GENYAL	Spain	221 (48%; 6-9y; 5% underweight, 16.3% overweight and 9.1% obese)	*BDNF-AS* rs925946 (G > T)	↓	Low	Neutral
Coffee	Muhammad et al. 2019		Indonesia	455 (51%; 19-56y; 25 ± 4.8-25.2 ± 5.5 kg/m^2^)	*UCP2* −866G > A	↓	Low	Neutral
	Wang et al. 2017	HPFS, NHS, WHI	US	20,605 (75%; 30-79y; 25.1 ± 4.9-25.8 ± 4.4 kg/m^2^)	Weighted (β) GRS: 77 SNPs	↓	Low	Neutral
Carbohydrate	Alathari et al. 2021	MINANG/GeNuIne	Indonesia	110 females (25-60; 24.1 ± 4.3-25.7 ± 4.4 kg/m^2^)	Unweighted GRS: 5 Vitamin D-related SNPs	↑	Low	Low
	Czajkowski et al. 2020		Polish	819 (53%; 18-79y; 28.5 ± 6.6 kg/m^2^)	*FTO* rs3751812 (G > T), rs8044769 (C > T) and rs8050136 (A > C)	↓	Low	Neutral
	Lim et al. 2014		Korea	1,128 (100%; 20-59y; 23.1 ± 0.2 kg/m^2^)	*APOA5* rs662799 (T > C)	↓	Low	Low
	Martínez et al. 2003		Spain	313 (79%; 20-60y; 50% obese with BMI ≥ 30 kg/m^2^)	*ADRB2* rs1042714 (C > G)	↑	Low	Neutral
	Smith et al. 2008	BPRHS	US	920 (72%; 45-74y; males 29.7 ± 5.3 and females 33.1 ± 7.1 kg/m^2^)	*PLIN* rs894160 (G > A)	↔	Low	Neutral
	Sonestedt et al. 2009	MDC	Sweden	4,839 males (NS; 44-74y; 25.5 kg/m^2^)	*FTO* rs9939609 (T > A)	↓	Low	Low
	Davis et al. 2010		US	153 (75%; 8-18y; 30.7 ± 8.6-32.5 ± 8.6 kg/m^2^)	*PNPLA3* rs738409 (C > G)	↑	Low	Low
	Goni et al. 2015		Spain	611 (78%; 50 ± 13y; 24 kg/m^2^)	Unweighted GRS: 16 SNPs	↑	Low	Low
	Rukh et al. 2017	MDCS	Sweden	4,800 (60%; 46-68y; 25.6 kg/m^2^)	*AMY1* copy number variants (CNV)	↑	Low	Low
	Vazquez-Moreno et al. 2020		Mexico	764 (53%; 6-12y; 16.9 ± 3.0-24.4 ± 2.7 kg/m^2^)	*AMY1A/AMY2A* copy numbers	↔	Low	Neutral
	Smith et al. 2008	BPRHS	US	920 (72%; 45-74y; males 29.7 ± 5.3 and females 33.1 ± 7.1 kg/m^2^)	*PLIN* rs2289487 (T > C), rs894160 (G > A), rs2304795 (A > G) and rs1052700 (A > T)	↔	Low	Neutral
	Smith et al. 2008	BPRHS	US	920 (72%; 45-74y; males 29.7 ± 5.3 and females 33.1 ± 7.1 kg/m^2^)	*PLIN* rs894160 (G > A)	↓	Low	Neutral
	Huriyati et al. 2020		Indonesia	261 (15-21y; 41% overweight and obese)	*KCNJ11* E23K (E > K)	↑	Low	Neutral
	Rocha et al. 2018	SCAALA	Brazil	1,211 (46%; 4-11y; 8.8% overweight and 4.6% obese)	*LEPR* rs1137100 (A > G); rs1177681 (G)	↑	Low	Low
	Rocha et al. 2018	SCAALA	Brazil	1,211 (46%; 4-11y; 8.8% overweight and 4.6% obese)	*LEPR* rs8179183 (G > C)	↓	Low	Low
Dairy products	Smith, Tucker, Arnett, et al. 2013	GOLDN, BPRHS	US	2,071 (61%; 45-74y; 28.0 ± 6.2-32.8 ± 6.9 kg/m^2^)	*APOA5* rs5082 (T > C)	↑	Low	Low
Dietary Diversity Score (DDS)	Goodarzi et al. 2021	TLGS	Iran	4,480 (39-67%; ≥18y; 27.1 ± 5.0 kg/m^2^)	*FTO* rs1121980 (C > T) and rs8050136 (A > G)	↓	Low	Neutral
	Goodarzi et al. 2021	TLGS	Iran	4,480 (39-67%; ≥18y; 27.1 ± 5.0 kg/m^2^)	*FTO* rs1421085 (T > C)	↔	Low	Neutral
Dietary Inflammatory Index (DII)	Yarizadeh et al. 2021		Iran	266 (100%; 18-56y; 30.3 ± 3.7 kg/m^2^)	*MC4R* rs17782313 (T > C)	↑	Low	Neutral
Dietary pattern (nuts, sweets, diary, wheat-based diets)	Zhu, Xue, Guo, Deng, et al. 2020	NISCOC	China	1,292 (49%; 7-12y; 19.4% obese)	*CMTM7* rs347134 (A > G)	↔	Low	Low
	Wang, Garcia-Bailo, et al. 2014	TNH	Canada	1,455 (68%; 20-29y; 23.3 ± 0.2-24.1 ± 0.4 kg/m^2^)	*ABO* rs8176719 (Del > G) and rs8176746 (A > G)	↔	Low	Neutral
Fiber	Goni et al. 2015		Spain	611 (78%; 50 ± 13y; 24 kg/m^2^)	Unweighted GRS: 16 SNPs	↓	Low	Low
	Hosseini-Esfahani, Koochakpoor, Daneshpour, Mirmiran, et al. 2017	TLGS	Iran	1,254 (34%; ≥18; 50% obese with BMI ≥ 30 kg/m^2^)	Weighted (OR) GRS: 6 *FTO* SNPs	↑	Low	Low
	Nakamura et al. 2016	Takahata	East Asian	1,620 (55%; >40y; 23.4 ± 3.1 kg/m^2^)	Weighted (β) GRS: 29 SNPs	↑	Neutral	Low
	Zhu, Xue, Guo, and Yang 2020	NISCOC	China	789 (50%; 7-12y; 21% obese)	*LMX1B* rs10733682 (G > A)	↓	Low	Low
Fish intake	Huang, Wang, Heianza, Wiggs, et al. 2019	NHS, HPFS, WHI, SCHS	US, Europe and Singapore	29,674 (70%; 30-75y; 23.4 ± 3.3-28.3 ± 5.5 kg/m^2^)	*FADS* rs174570 (C > T)	↑	Low	Low
	Huang, Wang, Heianza, Zheng, et al. 2019	NHS, HPFS, WHI	US	24,357 (72%; 30-75y; 25.9 ± 3.3-28.3 ± 5.5 kg/m^2^)	Weighted (β) GRS: 77 SNPs	↓	Low	Low
Fried foods	Livingstone, Celis-Morales, Navas-Carretero, et al. 2016	Food4me	Europe	1,277 (58%; 18-79y; 23.1 ± 0.2 kg/m^2^)	*FTO* rs9939609 (T > A)	↑	Low	Neutral
Health diet score (HDS) and Health diet index (HDI)	Nettleton et al. 2015	18 cohorts	US and Europe	68,317 (69%; 38 ± 5-75 ± 3y; 24.8 ± 4.5-28.2 ± 4.6 kg/m^2^)	Unweighted GRS: 3 SNPs; *GRB14* rs10195252 (T); *LINGO2/LRRN6C* rs10968576 (G); *LYPLAL11* rs4846567 (G); *MTIF3* rs4771122 (G)	↑	Low	Low
	Young, Wauthier, and Donnelly 2016	UK biobank	UK	119,132 (48%; 40-69y; 27.4 ± 4.8 kg/m^2^)	*FTO* rs1421085 (T > C)	↑	Low	Low
	Han et al. 2020	CARTaGENE biobank	Canada	6,087 (54%; 40-69y; 27.3 kg/m^2^)	Unweighted GRS: 97 SNPs	↓	Low	Low
	Mousavizadeh et al. 2020	TLGS	Iran	3,850 (63%; ≥18y; 44% obese with BMI ≥ 30 kg/m^2^)	*MC4R* rs12970134 (G > A) and rs17782313 (T > C)	↓	Low	Neutral
Legumes dietary pattern (LDP)	Mousavizadeh et al. 2020		Iran	265 (53%; 18-55y; NS)	*9p21* rs1333048 (A > C)	↑	Low	Low
Macronutrients including protein, fat and carbohydrate	Czajkowski et al. 2020		Polish	819 (53%; 18-79y; 28.5 ± 6.6 kg/m^2^)	*FTO* rs9939609 (T > A)	↔	Low	Neutral
	Nasreddine et al. 2019		Lebanese	308 (63%; ≥18y; 27.8 ± 5.6 kg/m^2^)	*FTO* rs1558902 (T > A) and rs9939609 (T > A)	↔	Low	Low
	Rukh et al. 2013	MDCS	Sweden	29,480 (60%; 58 ± 8y; 25.8 ± 4.1 kg/m^2^)	Unweighted GRS: 13 SNPs	↔	Low	Low
Meal skipping- Snacking, infrequent and unhealthy eating	Jaaskelainen et al. 2013	NFBC1986	Northern Finland	4,665 (53%; 16y; 21.2 ± 3.4 kg/m^2^)	Unweighted GRS: 8 SNPs; *FTO* rs1421085 (T > C); *MC4R* rs17782313 (T > C)	↑	Low	Low
	Masip et al. 2020	FinnTwin16 Study	Finland	3,977 (57%; 31-37y; 24.8 kg/m^2^)	Weighted GRS: 1,148,565 SNPs	↑	Low	Low
Meat-based diet	Zhang et al. 2015		China	1,042 (35%; 7-12y; 51% obese)	Copy number variants (CNV) 10q11.22	↑	Low	Neutral
Mediterranean diet (MD)	Barchitta et al. 2014		Italy	380 females (NS; 29y; 8.7% underweight, 20.5% overweight and 12.1% obese)	*TNFα* rs1800629 (G > A)	↔	Low	Low
	Hosseini-Esfahani, Koochakpoor, Daneshpour, Sedaghati-khayat, et al. 2017	TLGS	Iran	1,254 (34%; ≥18y; 50% obese with BMI ≥ 30 kg/m^2^)	Weighted (OR) GRS: 6 *FTO* SNPs	↓	Low	Low
	Seral-Cortes et al. 2020	HELENA	Europe	605 (52%; 11-19y; 21.1 kg/m^2^)	Unweighted GRS: 21 SNPs	↓	Low	Neutral
	Sotos-Prieto et al. 2020	BPRHS	US	1,120 (65-72%; 15-75y; 31.4 ± 6.5-32.3 ± 6.7 kg/m^2^)	*TCF7L2* rs12255372 (G > T)	↔	Low	Low
	Sotos-Prieto et al. 2020	BPRHS	US	1,120 (65-72%; 15-75y; 31.4 ± 6.5-32.3 ± 6.7 kg/m^2^)	Unweighted GRS: 2 *TCF7L2* SNPs; *TCF7L2* rs7903146 (C > T)	↓	Low	Low
Protein (plant and animal-based)	Alsulami, Aji, et al. 2020	MINANG/GeNuIne	Indonesia	110 females (25-60y; 25.13 ± 4.2 kg/m^2^)	Unweighted GRS: 15 SNPs	↑	Low	Low
	Ankarfeldt et al. 2014	MONICA, DCH and Inter99	Denmark	7,054 (49-52%; median age 41y; NS)	Unweighted GRS: 50 SNPs	↔	Low	Low
	Czajkowski et al. 2020		Polish	819 (53%; 18-79y; 28.5 ± 6.6 kg/m^2^)	*FTO* rs3751812 (G > T), rs8044769 (C > T) and rs8050136 (A > C)	↑	Low	Neutral
	Doo and Kim 2011	KoGES	Korea	3,090 males (40-59y; 24.5 ± 0.1 kg/m^2^)	*ESR1* rs1884051 (C > T)	↓	Low	Low
	Goni et al. 2015		Spain	611 (78%; 50 ± 13y; 24 kg/m^2^)	Unweighted GRS: 16 SNPs	↑	Low	Low
	Goni et al. 2015		Spain	611 (78%; 50 ± 13y; 24 kg/m^2^)	Unweighted GRS: 16 SNPs	↓	Low	Low
	Lim et al. 2014		Korea	1,128 females (20-59y; 23.1 ± 0.2 kg/m^2^)	*APOA5* rs662799 (T > C)	↑	Low	Low
	Merritt, Jamnik, and El-Sohemy 2018	TNH	Canada	1,491 (69%; 20-29y; 23.2 ± 0.2 kg/m^2^)	*FTO* rs1558902 (T > A)	↓	Low	Neutral
	Nakamura et al. 2016	Takahata	East Asian	1,620 (55%; >40y; 23.4 ± 3.1 kg/m^2^)	Weighted (β) GRS: 29 SNPs	↓	Neutral	Low
	Rukh et al. 2013	MDCS	Sweden	29,480 (60%; 58 ± 8y; 25.8 ± 4.1 kg/m^2^)	*BDNF* rs4923461 (G > A)	↑	Low	Low
	Zhu, Xue, Guo, and Yang 2020	NISCOC	China	789 (50%; 7-12y; 21% obese)	*LMX1B* rs10733682 (G > A)	↑	Low	Low
Salt (or sodium)	Lee, Kwon, and Park 2017		Korea	796 non-obese (50%; 12y; NS)	*NEDD4L* rs2288774	↑	Low	Low
	Lee, Kwon, and Park 2017		Korea	796 non-obese (50%; 12y; NS)	*RK4* rs1024323; *GRK4* rs1801058; *SLC12A3* rs11643718; *ACE* rs4341; *AGT* rs699; *GNB3* rs5443; *ADD1* rs4961; *CYP*11β2 rs1799998	↔	Low	Low
	Lv et al. 2015		China	2,977 (35%; 7-17y; 17.1 ± 2.3-26.9 ± 4.1 kg/m^2^)	*KCTD15* rs11084753 (A > G); *MAP2K5* rs2241423 (A > G); *SEC16B* rs543874 (G > A)	↑	Low	Low
	Lv et al. 2015		China	2,977 (35%; 7-17y; 17.1 ± 2.3-26.9 ± 4.1 kg/m^2^)	*MC4R* rs17782313 (T > C)	↔	Low	Low
	Young, Wauthier, and Donnelly 2016	UK biobank	UK	119,132 (48%; 40-69y; 27.4 ± 4.8 kg/m^2^)	*FTO* rs1421085 (T > C)	↑	Low	Low
Soy bean	Wang, Tang, et al. 2014		China	944 (75%; 39 ± 12-43 ± 11y; 33% obese with BMI ≥ 25 kg/m^2^)	Haplotype of *HMGCR* rs3846662 (T)-rs3846663 (C)	↓	Low	Neutral
Sweetened soft drinks (SSD)	Jiang et al. 2019		China	1,149 (NS; 10-12y; NS)	*FTO* rs9939609 (T > A)	↑	Neutral	Low
	Olsen et al. 2016	MONICA, DCH and Inter99	Denmark	4,765 (50%; 41y; NS)	Unweighted GRS: 50 SNPs	↑	Low	Neutral
	Rocha et al. 2018	SCAALA	Brazil	1,211 (46%; 4-11y; 8.8% overweight and 4.6% obese)	*LEPR* rs1137100 (A > G)	↑	Low	Low
Total energy intake	Celis-Morales et al. 2017	UK biobank	UK	48,170 (54%; 37-69y; 26.9 ± 4.5 kg/m^2^)	Weighted (β) GRS: 93 SNPs	↑	Low	Low
	Doo and Kim 2011	KoGES	Korea	3,090 males (40-59y; 24.5 ± 0.1 kg/m^2^)	*ESR1* rs1884051 (C > T)	↑	Low	Low
	Gong et al. 2021	CHNS	China	2,216 (60%; 50y; 24 kg/m^2^)	*BCL2* rs12454712 (G > A); *FTO* rs8050136 (A > C)	↑	Low	Neutral
	Gong et al. 2021	CHNS	China	2,216 (60%; 50y; 24 kg/m^2^)	*FTO* rs9939609; *BDNF* rs11030104 and rs6265; *TRHR* rs16892496 and rs7832552; *1p31* rs2568958; *TMEM18* rs7561317; *SEC16B* rs574367; *MC4R* rs12970134; *KCNQ1* rs2237892	↔	Low	Neutral
	Higashibata et al. 2016	J-MICC	Japan	4,023 (54%; 35-69y; 23.0 ± 3.0 kg/m^2^)	*SIRT1* rs1467568 (A > G) and rs7895833 (G > A)	↑	Low	Low
	Lee et al. 2021	KARE, CAVAS, HEXA	Korea	46,515 (52-64%; 40-79y; 37% obese with BMI ≥25 kg/m^2^)	Weighted (β) GRS: 62 SNPs	↔	Low	Low
	Lim et al. 2014		Korea	1,128 females (20-59y; 23.1 ± 0.2 kg/m^2^)	*APOA5* rs662799 (T > C)	↑	Low	Low
	Miyaki et al. 2005		Japan	295 males (46 ± 12y; 23.3 ± 3.3 kg/m^2^)	*ADRB3* rs4994 (T > C)	↑	Low	Low
	Park et al. 2016	KoGES	Korea	8,842 (52%; 40-69y; 44% BMI < 23, 21% BMI 23-25 and 35% BMI > 25)	*MC4R* rs17782313 (T > C)	↑	Low	Low
	Park et al. 2016	KoGES	Korea	8,842 (52%; 40-69y; 44% BMI < 23, 21% BMI 23-25 and 35% BMI > 25)	*MC4R* rs571312 (C > A)	↔	Low	Low
	Song et al. 2007		Japan	285 males (46 ± 12y; 23.3 ± 3.3 kg/m^2^)	*IL6R* Asp358Ala (T > G)	↑	Low	Low
Total dietary fat	Alsulami, Nyakotey, et al. 2020	MINANG/GeNuIne	Indonesia	302 (54%; 25-60y; 26.6 ± 5.0 kg/m^2^)	Unweighted GRS: 4 SNPs	↑	Low	Low
	Casas-Agustench et al. 2014	MESA and GOLDN	US and Europe	2,817 adults (NS; NS; 27.9-28.5 kg/m^2^)	Weighted (β) GRS: 63 SNPs	↑	Low	Low
	Celis-Morales et al. 2017	UK biobank	UK	48,170 (54%; 37-69y; 26.9 ± 4.5 kg/m^2^)	Weighted (β) GRS: 93 SNPs	↑	Low	Low
	Corella et al. 2007	FOS	New Zealand	2,280 adults (53%; NS; males 28.2 ± 4.0 and females 26.7 ± 5.5 kg/m^2^)	*APOA5* rs662799 (T > C)	↓	Low	Neutral
	Corella et al. 2007	FOS	New Zealand	2,280 adults (53%; NS; males 28.2 ± 4.0 and females 26.7 ± 5.5 kg/m^2^)	*APOA5* rs3135506 (C > G)	↔	Low	Neutral
	Czajkowski et al. 2020		Polish	819 (53%; 18-79y; 28.5 ± 6.6 kg/m^2^)	*FTO* rs3751812 (G > T) and rs8050136 (A > C)	↑	Low	Neutral
	Czajkowski et al. 2020		Polish	819 (53%; 18-79y; 28.5 ± 6.6 kg/m^2^)	*FTO* rs8044769 (C > T)	↓	Low	Neutral
	Dominguez-Reyes et al. 2015		Mexico	200 (42%; 18-25y; median 27.4 kg/m^2^)	*APOA5* rs3135506 (C > G); *LEPR* rs1137101 (A > G)	↑	Low	Neutral
	Dominguez-Reyes et al. 2015		Mexico	200 (42%; 18-25y; median 27.4 kg/m^2^)	*APOA2* −1730 G > T (rs3813627) and −265 T/C (rs5082); *APOA5* −1131 T > C (rs662799) and *LEPR* 668 A > G (rs1137101)	↔	Low	Neutral
	Doo, Won, and Kim 2015	KoGES	Korea	6,470 (51%; 40-59y; 24.5 ± 0.1 kg/m^2^)	*APOB* rs1469513 (A > G)	↑	Low	Low
	Hiroi et al. 2011		Japan	1,231 males (31-76y; 23.8 ± 2.8 kg/m^2^)	*PRDX3* rs1553850 (A > T), rs3377 (A > C), rs3740562 (A > G), rs7768 (G > C) and rs2271362 (C > T)	↑	Low	Neutral
	Huriyati et al. 2016		Indonesia	261 (NS; 16-18y; 41% obese)	*UCP2* −866G > A	↓	Neutral	Neutral
	Labayen et al. 2016	HELENA	Europe	652 (NS; 13-16y; 20.7 ± 3.2-22.3 ± 4.1 kg/m^2^)	*FTO* rs9939609 (T > A)	↑	Low	Neutral
	Lim et al. 2014		Korea	1,128 females (20-59y; 23.1 ± 0.2 kg/m^2^)	*APOA5* rs662799 (T > C)	↓	Low	Low
	Park et al. 2013	PAGE	US	36,990 adults (52%; NS; 25.7 kg/m^2^)	*FTO* rs8050136 (A > G)	↑	Low	Low
	Robitaille et al. 2003	Quebec Family Study	French-canadian	720 (57%; 18-64y; 26.9 ± 7.2-28.2 ± 7.0 kg/m^2^)	*PPARG* rs1801282 (C > G)	↑	Low	Neutral
	Robitaille, Houde, et al. 2007		French-canadian	351 (28%; 47 ± 11y; 28.1 ± 4.3 kg/m^2^)	*CPT1A* A275T (A > T); *CPT1B* -18C > T and p.E531K (E > K);	↑	Neutral	Neutral
	Robitaille, Perusse, et al. 2007	Quebec Family Study	French-Canadian	645 (57%; 18-64y; mean WC: 90.2 ± 18.8cm)	*GHRL* rs696217 (C > A); *GNB3* 825 C > T; *PPARG* rs1801282 (C > G)	↑	Neutral	Neutral
	Sanchez-Moreno et al. 2011		Spain	1,465 (NS; 20-65y; 25-40 kg/m^2^)	*APOA5* rs662799 (T > C)	↑	Neutral	Neutral
	Sonestedt et al. 2009	MDC	Sweden	4,839 males (44-74y; 25.5 kg/m^2^)	*FTO* rs9939609 (T > A)	↑	Low	Low
	Sonestedt et al. 2011	MDC	Sweden	22,799 (73%; 44-74y; NS)	*FTO* rs9939609 (T > A)	↑	Low	Low
	Nakamura et al. 2016	Takahata	East Asian	1,620 (55%; >40y; 23.4 ± 3.1 kg/m^2^)	Weighted (β) GRS: 29 SNPs	↓	Neutral	Low
Saturated fats (SFA)	Alsulami, Nyakotey, et al. 2020	MINANG/GeNuIne	Indonesia	302 (54%; 25-60y; 26.6 ± 5.0 kg/m^2^)	Unweighted GRS: 4 SNPs	↑	Low	Low
	Casas-Agustench et al. 2014	MESA and GOLDN	US and Europe	2,817 adults (NS; NS; 27.9-28.5 kg/m^2^)	Weighted (β) GRS: 63 SNPs	↑	Low	Low
	Celis-Morales et al. 2017	UK biobank	UK	48,170 (54%; 37-69y; 26.9 ± 4.5 kg/m^2^)	Weighted (β) GRS: 93 SNPs	↑	Low	Low
	Corella et al. 2009	FOS, GOLDN, BPRHS	US	3,462 (57%; 20-80y; NS)	*APOA2* rs5082 (T > C)	↑	Low	Low
	Corella et al. 2011	GOLDN and BPRHS	US	2,162 (53-71%; 45-75y; 22.7 ± 3.7-30.9 ± 5.1 kg/m^2^)	*FTO* rs1121980 (C > T) and rs9939609 (T > A)	↑	Low	Low
	Dominguez-Reyes et al. 2015		Mexico	200 (42%; 18-25y; median 27.4 kg/m^2^)	*APOA2* −1730 G > T (rs3813627) and −265 T/C (rs5082); *APOA5* −1131 T > C (rs662799) and *LEPR* 668 A > G (rs1137101)	↔	Low	Neutral
	Dominguez-Reyes et al. 2015		Mexico	200 (42%; 18-25y; median 27.4 kg/m^2^)	*APOA5* rs3135506 (C > G); *LEPR* rs1137101 (A > G)	↑	Low	Neutral
	Garske et al. 2019	UK biobank	Europe	167,908 (NS)	*CARM1* rs112438892; *GLTSCR2– SNORD23* rs17625418; *PLIN2* rs882881 and rs867773; *RDH8–COL5A3* rs35678764; *LIPE–LIPE-AS1* rs10422283	↑	Low	Low
	Garske et al. 2019	UK biobank	Europe	167,908 (NS)	*GLTSCR2– SNORD23* rs1974817; *RGMB* rs58631862; *SH3GL3* rs74249860; *HOOK2–JUNB* rs3848589; *RNU2-10P* rs35213231; *BLVRB– SPTBN4* rs41322049; *LDB3* rs10788522; *TNFRSF1B–* MIR4632 1:12245360_CCTTTTT_C	↓	Low	Low
	Goni et al. 2015		Spain	611 (78%; 50 ± 13y; 24 kg/m^2^)	Unweighted GRS: 16 SNPs	↑	Low	Low
	Nasreddine et al. 2019		Lebanese	308 (63%; ≥18y; 27.8 ± 5.6 kg/m^2^)	*TCF7L2* rs7903146 (C > T)	↓	Low	Low
	Robitaille et al. 2003	QueÂbec Family Study	French-canadian	720 (57%; 18-64y; 26.9 ± 7.2-28.2 ± 7.0 kg/m^2^)	*PPARG* rs1801282 (C > G)	↑	Low	Neutral
	Smith, Tucker, Lee, et al. 2013	BPRHS	US	920 (72%; 45-74y; 31.9 ± 6.6 kg/m^2^)	*LRP1* rs1799986 (C > T)	↑	Low	Low
	Smith, Tucker, Lee, et al. 2013	BPRHS	US	920 (72%; 45-74y; 31.9 ± 6.6 kg/m^2^)	*LRP2* rs715948 (C > T) and rs1800191 (G > A)	↔	Low	Low
Mono- unsaturated fats (MUFA)	Alsulami, Nyakotey, et al. 2020	MINANG/GeNuIne	Indonesia	302 (54%; 25-60y; 26.6 ± 5.0 kg/m^2^)	Unweighted GRS: 4 SNPs	↑	Low	Low
	Corella et al. 2007	FOS	New Zealand	2,280 adults (53%; NS; males 28.2 ± 4.0 and females 26.7 ± 5.5 kg/m^2^)	*APOA5* rs662799 (T > C)	↓	Low	Neutral
	Garaulet et al. 2014		Spanish and North American	2,214 (50-82%; 40 ± 12-48 ± 16y; 28.3 ± 5.6-31.1 ± 5.4 kg/m^2^)	*REV-ERB-ALPHA1* rs2314339 (A > G)	↔	Low	Neutral
	Riedel et al. 2013	ALSPAC	Europe	2,346 (49%; 9y; mean FMI: 4.1 ± 2.2 kg/m^2^)	Unweighted GRS: 8 SNPs	↓	Low	Neutral
	Warodomwichit et al. 2009	GOLDN	US	1,083 (52%; 17-92y; 28.7 ± 0.3 kg/m^2^)	*ADIPOQ* − 11377C > G	↔	Low	Neutral
	Warodomwichit et al. 2009	GOLDN	US	1,083 (52%; 17-92y; 28.7 ± 0.3 kg/m^2^)	*ADIPOQ* − 11391G > A	↓	Low	Neutral
Poly-unsaturated fats (PUFA)	Alsulami, Nyakotey, et al. 2020	MINANG/GeNuIne	Indonesia	302 (54%; 25-60y; 26.6 ± 5.0 kg/m^2^)	Unweighted GRS: 4 SNPs	↑	Low	Low
	Dumont et al. 2018	MONALISA	French	3,069 (50%; 35-74y; 26.1 ± 5 kg/m^2^)	*FADS1* rs174547 (T > C)	↑	Low	Low
	Goni et al. 2015		Spain	611 (78%; 50 ± 13y; 24 kg/m^2^)	Unweighted GRS: 16 SNPs	↓	Low	Low
	Junyent et al. 2010	GOLDN	US	936 (52%; 33-65y; 28.7 ± 4.7 kg/m^2^)	*ADAM17* rs10495563 (A > G)	↑	Low	Neutral
	Junyent et al. 2010	GOLDN	US	936 (52%; 33-65y; 28.7 ± 4.7 kg/m^2^)	*ADAM18* rs11684747 (A > G), rs1880439 (C > A), rs1056204 (A > C), rs34367192 (C > T) and rs4622692 (G > T)	↔	Low	Neutral
	Ma et al. 2014	BPRHS and ARIC	US	12,046 (54-72%; 44-75y’26.5-32.9 kg/m^2^)	*LPL* rs2083637 (A > G), rs17411031 (C > G), rs13702 (T > C) and rs2197089 (A > G)	↔	Low	Low
	Ma et al. 2014	BPRHS and ARIC	US	12,046 (54-72%; 44-75y’26.5-32.9 kg/m^2^)	*LPL* rs320 (T > G)	↓	Low	Low
	Riedel et al. 2013	ALSPAC	Europe	2,346 (49%; 9y; mean FMI: 4.1 ± 2.2 kg/m^2^)	Unweighted GRS: 8 SNPs; *LEPR* rs1137100 (A > G)	↓	Low	Neutral
	Chen et al. 2019	GLACIER	Sweden	5,160 adults (61%; NS; 25.5 ± 3.8 kg/m^2^)	*FADS* rs174570, rs174602, rs74771917, rs3168072, rs12577276 and rs7115739	↔	Low	Low
n-3 PUFA	Huang, Wang, Heianza, Wiggs, et al. 2019	NHS, HPFS, WHI, SCHS	US, Europe and Singapore	29,674 (70%; 30-75y; 23.4 ± 3.3-28.3 ± 5.5 kg/m^2^)	*FADS* rs174570 (C > T)	↑	Low	Low
	Huang, Wang, Heianza, Zheng, et al. 2019	NHS, HPFS, WHI	US	24,357(72%; 30-75y; 25.9 ± 3.3-28.3 ± 5.5 kg/m^2^)	Weighted (β) GRS: 77 SNPs	↓	Low	Low
	Huang, Wang, Heianza, Wiggs, et al. 2019	NHS, HPFS, WHI, SCHS	US, Europe and Singapore	29,674 (70%; 30-75y; 23.4 ± 3.3-28.3 ± 5.5 kg/m^2^)	*FADS* rs174602 (T > C), rs7115739 (T > A)	↔	Low	Low
	Joffe et al. 2014		South American	268 female adults (NS)	*IL-6* rs1800795 (G > C); *IVS3* rs1554606 (G > T); *IVS4* rs2069845 (A > G)	↓	Low	Low
	Lemas et al. 2013	CANHR	Alaska	1,073 (52%; ≥14y; NS)	Unweighted GRS: 10 SNPs from 10 genes	↑	Low	Low
	Vaughan et al. 2015	CANHR	Alaska	982 (52%; ≥14y; 25.6 kg/m^2^)	*BTBD10* rs730414 (G > T)	↓	Low	Neutral
n-6 PUFA	Bauman-Fortin et al. 2019	THN	Canada	898 (72%; 20-29y; 18.5-24.9 kg/m^2^)	*NFKB1* rs11722146 (G > A), rs13117745 (C > T), rs1609798 (C > T), rs4648022 (C > T), rs4648090 (A > G), rs1599961 (A > G), rs230511 (A > G), rs7674640 (C > T) and rs3774932 (A > G)	↔	Low	Low
	Joffe et al. 2014		South American	268 female adults (NS)	*IVS4* rs2069845 (A > G); *IL-6* rs1800795 (G > C); *IVS3* rs1554606 (G > T)	↑	Low	Low
	Nieters, Becker, and Linseisen 2002	EPIC	Europe	308 (62%; 35-65y; 50% obese with BMI ≥35 kg/m^2^)	*LEP* rs7799039 (G > A)	↓	Low	Neutral
	Nieters, Becker, and Linseisen 2002	EPIC	Europe	308 (62%; 35-65y; 50% obese with BMI ≥35 kg/m^2^)	*PPARA* rs1800206 (C > G) and Exon 6 (C > T); *UCP1* −3826 (A > G), *UCP2* 45bp exon (del/ins), *UCP3*–55C/T, *BAR-2* Arg16Gly (G > A) and Gln27Glu (C > G), *APM1* Gln27Glu (T > G), *SORBS1* Thr228Ala (A > G), *HSL* − 60 G/C	↔	Low	Neutral
	Nieters, Becker, and Linseisen 2002	EPIC	Europe	308 (62%; 35-65y; 50% obese with BMI ≥35 kg/m^2^)	*PPARG* rs1801282 (C > G); *TNFα* G307A	↑	Low	Neutral
Vitamin A	Galmes, Palou, and Serra 2020		Spain	158 males (37 ± 17y; 26.5 ± 5.0 kg/m^2^)	Unweighted GRS: Haplotype-*SCARB1* rs5888 (C > T); *UCP2* rs659366 (G > A); *UCP1* rs1800629 (C > T)	↓	Low	Neutral
	Goodwin et al. 2015		French-Canadian	947 (52%; 12-18y; NS)	*RBP4* rs10882272 (C > T)	↓	Low	Low
Vitamin B2, folate, B12	Huang et al. 2015	NHS and HPFS	US	14,870 (55%; 30-75; 25.0 ± 4.7-25.7 ± 3.2 kg/m^2^)	*HIF3A* rs3826795 (G > A)	↓	Low	Low
	Li et al. 2019	WHI	US	5,687 females (65-79y; 28.3 ± 5.5 kg/m^2^)	*SREBF1* rs752579 (T > C)	↑	Low	Low
Vitamin C	Larsen et al. 2014a	MONICA, DCH and INTER99	Denmark	7,569 (49-52%; 31-61y; NS)	Unweighted GRS: 50 SNPs	↔	Low	Low
Vitamin E	Mansego et al. 2015	Hortega	Spain	738 (47%; 61 ± 17y; 33% with abdominal obesity)	*COMT* rs740603 (G > A); *TXN* rs2301241 (T > C)	↓	Low	Low
Western diet	Hosseini-Esfahani et al. 2019	TLGS	Iran	4,292 (56%; ≥18y; NS)	Weighted (OR) GRS: 6 *FTO* SNPs	↑	Low	Low

Outcome measures ^a^include body weight, BMI, waist circumference and adiposity.

Risk of bias (general) ^1^was assessed by Quality Criteria Checklist and risk of bias (genetic) ^2^was assessed by a methodology quality evaluation method specifically tailored to gene-diet interaction research.

↓ indicates significant decrease in body weight and adiposity; ↑indicates significant increase in body weight and adiposity; ↔ indicates no significant differences between the risk alleles and non-risk alleles carriers.

AHEI-2010, Alternative Healthy Eating Index; AMED, Alternate Mediterranean Diet; BMI, body mass index; DASH, Dietary Approaches to Stop Hypertension; GRS, genetic risk score; NS, not stated; SD, standard deviation.

### Data synthesis

Tables were constructed to synthesize the evidence of gene-diet interactions on nutritional status based on the study design and measure outcomes, and were ordered based on the types of dietary components or interventions. GraphPad Prism 9 was used to generate a Forest plot (without calculating a summary measure given high heterogeneity of the studies) to summarize the interactions between genetic risk score (GRS), dietary components and BMI. GRS is defined the weighted sum of an individual’s trait-associated alleles which derived from genome-wide associated study (GWAS) data.

## Results

**Figure 1.**
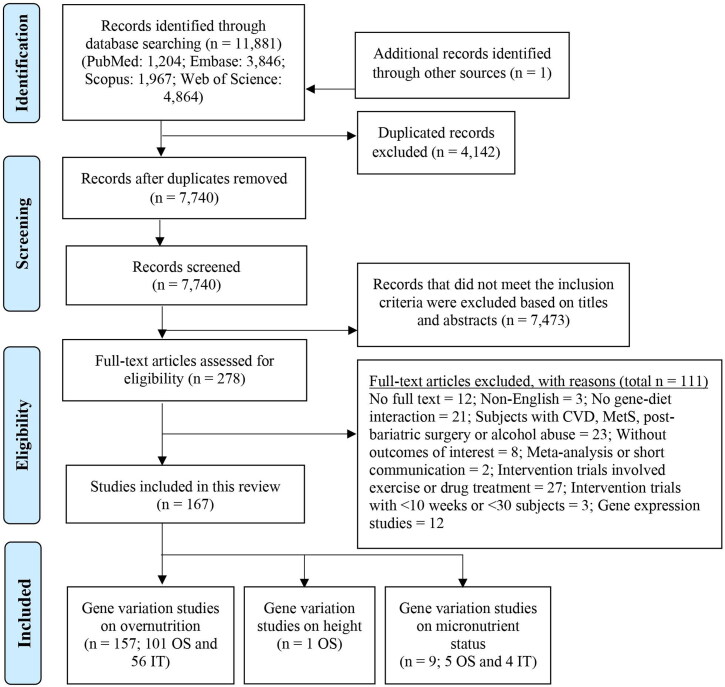
PRISMA flow diagram of identification and selection of studies. OS; observational studies, IT; intervention trials, CVD; cardiovascular diseases, MetS; metabolic syndrome

As shown in Figure 1, the literature search generated 11,881 records from four databases, with an additional article found through the citation lists. After removing duplicates, 7,740 articles were screened by title and abstract, and 278 full-text articles were assessed for eligibility. Of those, 167 articles were included in this review, which were comprised of: 157 articles (n = 101 observational studies and n = 56 intervention trials) that had investigated the interactions between genetic variants and dietary components on obesity; one article that reported on height; and 9 articles that reported on micronutrient status (n = 5 observational studies and n = 4 intervention trials). A few intervention trials (n = 12) investigated the interaction between weight loss outcomes and gene expression, but as beyond the scope of this review will not be discussed.

Study participants were recruited from 27 different populations, mainly from North and South American (e.g., the United States (US), including Alaska and Puerto Rican, Canada, Mexico, and Colombia) and Europe (the United Kingdom (UK), Spain, Italy, Denmark, Brazil, Sweden, the Netherland, France, Poland, Greece, Belgium and Finland). Whereas, a smaller number of studies were reported from the Middle East (Iran, Israel and Lebanon), East Asian (Korea, Japan, China), Southeast Asian (Singapore, Malaysia and Indonesia), and the Southwestern Pacific Ocean (New Zealand) populations. The age of participants ranged from 0 to 92 years, and the sample sizes ranged from 110 to 119,132 for observational studies and from 32 to 1,852 for intervention trials.

### Risk of bias

Based on the Quality Criteria Checklist, 101 observational and 26 intervention studies were assessed as having low risk of bias, whereas 6 observational and 34 intervention studies received a neutral rating (Table S3). The reasons for lower quality ratings for intervention trials were typically: lack of blinding procedure, non-randomization, inappropriate statistical analysis (e.g. without intention-to-treat analysis or no adjustment for baseline values or confounding factors), and no detailed description for withdrawal. In addition, the evaluation of methodological quality specifically designed for gene-diet interaction research found 97, 69 and 1 studies as having low, medium and high risk of bias, respectively (Table S3). The reasons for score reductions were mainly due to studies’ lack of: power analysis or insufficient sample size, application of Hardy-Weinberg Equilibrium and correction for multiple testing.

**Table 3.** Summary of intervention trials (n = 56) examining gene-diet interactions and weight loss outcomes.

Dietary interventions	Reference	Population	Name of trials	Study duration and design	Sample size (female %; age in years)	SNPs (alleles) or GRS	Weight loss outcome^a^	Risk of bias (general)^1^	Risk of bias (genetic)^2^
Doenjang	Cha et al. 2014	Korea		12w RCT	51 (84%; 19-65y)	*PPARG2* C1431T (C > T)	↓	Low	Neutral
	Lee et al. 2012	Korea		12w RCT	102 (86%; 35 ± 10-43 ± 12y)	*UCP1*	↓	Low	Neutral
Low-calorie diets (deficit of 500-600 kcal/day or 1200-1500kcal/day	Abete et al. 2009	Spain		8w pre and post	170 (47%; 20-50y)	*LEPR* Lys109Arg (A > G)	↓	Neutral	Low
	de Luis et al. 2014	Spain		3m pre and post	91 (79%; 49 ± 12y)	*GLP1R* rs6923761 (G > A)	↔	Neutral	Neutral
	de Luis, Fernández Ovalle, et al. 2018	Spain		3m pre and post	80 (67-75%; 47 ± 9y)	*BDNF* rs10767664 (A > T)	↓	Neutral	Neutral
	Hamada et al. 2011	Japan		2m pre and post	32 female adults (NS)	*ACE* insertion > deletion	↑	Neutral	Neutral
	Hamada et al. 2011	Japan		2m pre and post	32 female adults (NS)	*AT2R* or AGTR2 3123C > A	↔	Neutral	Neutral
	Heianza et al. 2017	US	POUND LOST	2y RCT	692 (61%; 30-70y)	*AMY1-AMY2* rs11185098 (G > A)	↓	Low	Low
	Hernandez-Guerrero et al. 2018	Mexico		3m pre and post	92 females (18-65y)	*CAT* rs7943316 (A > T) and rs1001179 (C > T); *GPX1* rs1050450 (C > T); *SOD1* rs2070424 (A > G)	↔	Neutral	High
	Hernandez-Guerrero et al. 2018	Mexico		3m pre and post	92 females (18-65y)	*SOD2* rs4880 (C > T)	↓	Neutral	High
	Labayen et al. 2015	Spain		12w pre and post	77 females (19-49y)	*MC4R* rs17782313 (T > C) and *FTO* rs9939609 (T > A)	↔	Neutral	Neutral
	Mammes et al. 2001	France		2.5m pre and post	179 adults (64%; NS)	*LEP* Asp96Asp (A > G), Lys109Arg (A > G), Gln223Arg (A > G), Ser343Ser (T > C), Lys656Asn (G > C), A + 37C (A>), Pro1019Pro (G > A)	↔	Neutral	Neutral
	Mammes et al. 2001	France		2.5m pre and post	179 adults (64%; NS)	*LEP* Ser343Ser (T > C)	↓	Neutral	Neutral
	Martinez-Lopez et al. 2013	Mexico		2m pre and post	109 (82%; 18-65y)	*FABP2* Ala54Thr	↓	Neutral	Low
	Matsuo et al. 2009	Japan (women)		14w pre and post	95 females (24-66y)	*PPARG* rs1801282 (C > G) and rs2292101 (C > T)	↔	Neutral	Low
	Matsuo et al. 2009	Japan (women)		14w pre and post	95 females (24-66y)	*PPARG* rs2959272 (G > T), rs1386835 (A > G), rs709158 (A > G), rs1175540 (C > A), rs1175544 (C > T), and rs1797912 (A > C)	↓	Neutral	Low
	Matsuo et al. 2012	Japan (women)		14w pre and post	204 females (24-66y)	*FTO* rs9939609 (T > A)	↑	Neutral	Low
	Nikpay et al. 2020	Canada		26w pre and post	1,852 adults (72-77%; NS)	*SGCG* rs679482 (C > A)	↑	Neutral	Low
	Ruiz et al. 2011	Spain		12w pre and post	78 females (19-49y)	*PLIN1* rs894160 (G > A)	↑	Neutral	Neutral
	Ruiz et al. 2011	Spain		12w pre and post	78 females (19-49y)	*PLIN1* rs2304795 (A > G)	↔	Neutral	Neutral
	Teixeira et al. 2020	Brazil		9-w pre and post	137 females (≥20y)	*ABCA1* rs1800977 (G > A)	↓	Neutral	Low
	Teixeira et al. 2020	Brazil		9-w pre and post	137 females (≥20y)	*ABCA1* rs2230806 (C > T), *ABCA7* rs2279796 (G > A) and *ABCG1* rs692383 (G > A) and rs3827225 (G > A)	↔	Neutral	Low
	Tsuzaki et al. 2009	Japan		2m pre and post	32 females (50 ± 8y)	*ADIPOQ* G276T (G > T)	↓	Neutral	Neutral
	Tsuzaki et al. 2009	Japan		2m pre and post	32 females (50 ± 8y)	*ADIPOQ* T45G, I164T and C-11377G	↔	Neutral	Neutral
Low-calorie diets with licorice extract	Namazi et al. 2017	Iran		8w RCT	72 (NS; 20-50y)	*PPARG* rs1801282 (C > G)	↔	Low	Neutral
Low-calorie diets and extra virgin oil	Rodrigues, Rosa, and Silveira 2018	Brazil	DieTBra Trial	12-w RCT	149 (85%; 18-65y)	*PPARG* Pro12Ala or rs1801282 (C > G)	↓	Low	Low
Low-calorie diets and extra virgin oil	Rodrigues, Rosa, and Silveira 2018	Brazil	DieTBra Trial	12-w RCT	149 (85%; 18-65y)	*IL6* G174C or rs1800795 (G > C)	↔	Low	Low
Low-carbohydrate diets (12%-45% of energy from carbohydrate)	Huang et al. 2018	US and Israel	POUND LOST and DIRECT	2y RCT	892 (53%; 30-70y)	*HNF1A* rs7957197 (A > T)	↓	Low	Low
	Lin et al. 2015	US	POUND LOST	2y RCT	723 (61%; 30-70y)	*NPY* rs16147 (C > T)	↓	Low	Low
	Seip et al. 2008	US		12w RCT	163 (39%; <20-70y_	*AGTR2* rs5950584 (G > T)	↑	Low	Neutral
	Stocks et al. 2012	Europe	NUGENOB and DiOGenes	10w/8w RCT	1,350 (65-75%; 20-50; 18-65y)	*TFAP2B* rs987237 (A > G)	↓	Low	Low
	Svendstrup et al. 2018	Caucasian	NUGENOB	10w RCT	707 (75%; 20-50y)	Unweighted GRS:47 SNPs; *VEGFA* rs1358980 (C > T)	↑	Low	Low
Low-fat diets (20-26% energy from fat)	de Luis, Aller, Izaola, de la Fuente, et al. 2012	Spain		3m RCT	305 (74%; 44 ± 15y)	*FTO* rs9939609 (T > A)	↔	Neutral	Low
	Goni et al. 2018	Spain		16w RCT	147 adults (67%; NS)	*ADCY3* rs10182181 (A > G)	↓	Low	Low
	Goni et al. 2019	US	POUND LOST	2y RCT	811 (NS; 30-70y)	*MTNR1B* rs10830963 (C > G)	↓	Low	Low
	Grau et al. 2009	Europe	NUGENOB	3w RCT	771 (75%; 20-50y)	*FTO* rs9939609 (T > A)	↔	Low	Low
	Grau et al. 2010	Europe	NUGENOB	10w RCT	771 (75%; 20-50y)	*TCF7L2* rs7903146 (C > T)	↓	Low	Low
	Heianza et al. 2016	US	POUND LOST	2y RCT	715 (61%; 30-70y)	*FGF21* rs838147 (T > C)	↓	Low	Low
	Li et al. 2020	US	POUND LOST	2y RCT	692 (61%; 51 ± 9-52 ± 8y)	Weighted (β)GRS: 5 SNPs	↓	Low	Low
	Mattei et al. 2012	US	POUND LOST	2y RCT	591 (59%; 30-70y)	*TCF7L2* rs12255372 (G > T)	↓	Low	Low
	Mattei et al. 2012	US	POUND LOST	2y RCT	591 (59%; 30-70y)	*TCF7L2* rs7903146 (C > T)	↑	Low	Low
	Ramos-Lopez et al. 2019	Spain		6w RCT	201 adults (NS)	Weighted (β) GRS: 95 SNPs from 59 genes	↓	Low	Neutral
	Seip et al. 2008	US		12w RCT	163 (39%; <20-70y)	*HNMT* rs12691940 (G > A); *PFKL* rs2838549 (G > A); *RARB* rs322695 (G > A)	↓	Low	Neutral
	Stocks et al. 2012	Europe	NUGENOB and DiOGenes	10w/8w RCT	1,350 (65-75%; 20-50; 18-65y)	*TFAP2B* rs987237 (A > G)	↓	Low	Low
Low-SFA and cholesterol diets	Xinli et al. 2007	China		3m non-RCT	83 (34%; 8-11y)	*ADRβ3* rs4994 (T > C)	↓	Neutral	Low
High-MUFA diets (67.5% of total fat from MUFA)	de Luis, Aller, Izaola, Conde, et al. 2013	Spain		3m pre and post	99 (80%; 49 ± 12y)	*FAAH* rs32440 (C > A)	↓	Neutral	Neutral
	de Luis, Aller, Izaola, Sagrado, et al. 2013	Spain		3m pre and post	122 (79%; 48 ± 12y)	*FABP2* Ala54Thr	↔	Neutral	Neutral
High-protein diets (25-40% energy from protein)	de Luis et al. 2015	Spain		9m RCT	284 (74%; 52 ± 11y)	*ADRB3* rs4994 (T > C)	↔	Neutral	Neutral
	de Luis et al. 2016a	Spain		9m RCT	283 (74%; 20-65y)	*TNFα* rs1800629 (G > A)	↔	Neutral	Neutral
	de Luis et al. 2016b	Spain		9m RCT	283 adults (72-78%; NS)	*UCP3* rs1800849 (C > T)	↓	Neutral	Neutral
	Ramos-Lopez et al. 2019	Spain		6w RCT	201 adults	Weighted (β) GRS: 95 SNPs from 59 genes	↓	Low	Neutral
	Tan and Mitra 2020	Malaysia		6m RCT	129 (84%; ≥18y)	Weighted (OR) GRS: 5 SNPs	↔	Low	Low
	Zhang et al. 2012	US	POUND LOST	2y RCT	742 (61%; 30-70y)	*FTO* rs9939609 (T > A)	↓	Low	Low
High-PUFA diets (22.7% total fat from PUFA)	de Luis, Aller, Izaola, Sagrado, et al. 2012	Spain		3m pre and post	111 (75%; 50 ± 10y)	*FABP2* Ala54Thr	↓	Neutral	Neutral
	de Luis, Izaola, et al. 2013	Spain		3m pre and post	99 (80%; 47 ± 10y)	*FAAH* rs324420 (C > A)	↔	Neutral	Neutral
Mediterranean diet	de Luis, Izaola, et al. 2018	Spain		12w pre and post	80 (75%; 26-65y)	*MTNR1B* rs10830963 (C > G)	↓	Neutral	Neutral
	Di Renzo et al. 2013	Italy		12w pre and post	56 (66%; 44 ± 14-47 ± 12y)	*MTHFR* C677T (C > T)	↑	Neutral	Neutral
	Di Renzo et al. 2018	Italy		4-w RCT	188 (NS; ≥16y)	*FTO* rs9939609 (T > A)	↓	Low	Low
	San-Cristobal et al. 2017	Europe	Food4me	6m RCT	1,263 (57%; 41 ± 13y)	Unweighted GRS: 14 SNPs	↓	Low	Low
	de Luis et al. 2019	Spain		3m pre and post	82 (75%; 26-61y)	*ADIPOQ* rs1501299 (T > A)	↔	Neutral	Neutral
Mediterranean diet (MD) (with VOO and nuts)	Razquin et al. 2010	Europe	PREDIMED	3y RCT	776 (55%; 55-80y)	*FTO* rs9939609 (T > A)	↓	Low	Low
Nutrigenetic guided diet	Frankwich et al. 2015	US	MOVE!	8w RCT	51 (NS; 48 ± 3-55 ± 3y)	*FTO* rs9939609; *APOA2* rs5082; *ADIPOQ* rs17300539; *KCTD10* rs10850219; *LIPC* rs1800588; *MMAB* rs2241201 and *PPARG* rs1801282	↓	Low	Neutral
Very low-calorie diets (∼500kcal/day)	Cha et al. 2006	Korea		1m pre and post	214 females (29 ± 9y)	*UCP3* Haplotype 1 (Ht1) and Tyr210Tyr	↓	Neutral	Neutral
	Cha et al. 2006	Korea		1m pre and post	214 females (29 ± 9y)	*UCP3* Int3-47G/A	↑	Neutral	Neutral
	Cha et al. 2007	Korea		1m pre and post	386 females (27 ± 1y)	*UCP2* Ala55Val	↑	Neutral	Low
	Rauhio et al. 2013	Finland		3m pre and post and 9m maintenance	75 females (25-45y)	*FTO* rs9939609 (T > A) and *ADRB2* rs1042714 (C > G)	↔	Low	Neutral
	Soenen et al. 2009	Belgium		6w pre and post and 1y follow up	118 (64%; 20-65y)	*PLIN1* rs2289487 (T > C)- *PLIN4* rs894160 (G > A)-haplotype; *PLIN5* rs2304795 (A > G) and *PLIN7* rs2304796 (G > A)-haplotype; *PLIN6* rs1052700 (A > T)	↓	Neutral	Neutral
	Verhoef et al. 2014	The Netherland		8w pre and post and 3-m maintenance	150 (74%; 20-50y)	Unweighted GRS: 6 SNPs	↓	Neutral	Low
	Yoon et al. 2007	Korea		1m pre and post	458 females (29 ± 9y)	*UCP2* 866G > A	↑	Neutral	Neutral
	Yoon et al. 2007	Korea		1m pre and post	458 females (29 ± 9y)	*UCP2* -1957G > A, +4787C > T, +7941 45bp-insdel; and UCP3 -35C > T, +2564G > C, +2877T > C, +3106G > A, +3854C > T, +4589T > C	↔	Neutral	Neutral
	Yoon et al. 2007	Korea		1m pre and post	458 females (29 ± 9y)	*UCP2*-*UCP3*-ht1	↓	Neutral	Neutral

Weight loss outcomes ^a^include post-intervention changes on body weight, body mass index, waist circumference and adiposity.

Risk of bias (general) ^1^was assessed by Quality Criteria Checklist and risk of bias (genetic) ^2^was assessed by a methodology quality evaluation method specifically tailored to gene-diet interaction research

↓ indicates significant greater reduction in weight loss outcomes among the risk alleles carriers; ↑indicates significant smaller reduction in weight loss outcomes; ↔ indicates no significant post-intervention changes in weight loss outcomes between the risk alleles and non-risk alleles carriers.

GRS, genetic risk score; MUFA, monounsaturated fats; NS, not stated; PUFA, polyunsaturated fats; RCT, randomized controlled trials; SFA, saturated fats; SNPs, single nucleotide polymorphisms.

### Observational studies: Gene-diet interactions and weight status

The evidence for the effects of gene-diet interactions on weight status from observational studies in the context of overnutrition (n = 101) are summarized in Table 2. We identified only one study reporting the effect of gene-diet interaction on undernutrition, assessed by body height. The authors reported that children in Greece carrying the risk allele (A allele) of insulin-like growth factor II (*IGF*) rs680, who consumed high intake of dairy product were taller compared to those with low intake of dairy product (Dedoussis et al. 2010).

The most investigated dietary components were dietary fats including total fat, saturated fatty acids (SFA), monounsaturated fatty acids (MUFA), total polyunsaturated fatty acids (PUFA), n-3 and n-6 PUFA, as well as dietary patterns that were assessed using scoring systems or *a posteriori* approaches. The majority of the studies reported that increased intakes of total fat (Alsulami, Nyakotey, et al. 2020; Celis-Morales et al. 2017; Czajkowski et al. 2020; Dominguez-Reyes et al. 2015; Doo, Won, and Kim 2015; Hiroi et al. 2011; Labayen et al. 2016; Park et al. 2013; Robitaille et al. 2003; Robitaille, Houde, et al. 2007; Robitaille, Perusse, et al. 2007; Sanchez-Moreno et al. 2011; Sonestedt et al. 2011; Sonestedt et al. 2009), SFA (Alsulami, Nyakotey, et al. 2020; Casas-Agustench et al. 2014; Celis-Morales et al. 2017; Corella et al. 2009; Corella et al. 2011; Dominguez-Reyes et al. 2015; Goni et al. 2015; Garske et al. 2019; Robitaille et al. 2003; Smith, Tucker, Lee, et al. 2013) and n-6 PUFA (Joffe et al. 2014; Nieters, Becker, and Linseisen 2002) were associated with increased risk of obesity among the individuals who had higher GRS or carrying the risk alleles of the genetic variants. Whereas increased intakes of MUFA (Corella et al. 2007; Garaulet et al. 2014; Riedel et al. 2013; Warodomwichit et al. 2009) and total PUFA or n-3 PUFA (Goni et al. 2015; Huang, Wang, Heianza, Zheng, et al. 2019; Joffe et al. 2014; Lemas et al. 2013; Ma et al. 2014; Riedel et al. 2013; Rocha et al. 2018; Vaughan et al. 2015) were associated with lower risk of obesity.

With respect to dietary patterns, studies showed those who either had higher GRS or were carrying the risk alleles of the genetic variants examined had unfavorable outcomes on body weight, BMI or adiposity when following unhealthy dietary patterns. This included meal skipping, snacking and diets with high inflammatory index, as well as meat-based diets and Western diets (Hosseini-Esfahani et al. 2019; Jaaskelainen et al. 2013; Masip et al. 2020; Wang, Tang, et al. 2014; Yarizadeh et al. 2021; Zhang et al. 2015; Zhu, Xue, Guo, Deng, et al. 2020). On the other hand, increased adherence to healthy dietary patterns rich in vegetables, fruits and whole grains was more likely to be associated with lower BMI or body fat (Barchitta et al. 2014; Ding et al. 2018; Goodarzi et al. 2021; Han et al. 2020; Hosseini-Esfahani, Koochakpoor, Daneshpour, Sedaghati-khayat, et al. 2017; Mollahosseini et al. 2020; Mousavizadeh et al. 2020; Nettleton et al. 2015; Seral-Cortes et al. 2020; Sotos-Prieto et al. 2020; Wang et al. 2018; Young, Wauthier, and Donnelly 2016). These studies included examination of dietary patterns such as the Mediterranean Diet (MD), the Alternate Mediterranean Diet (AMED), and the Dietary Approaches to Stop Hypertension (DASH) diet, or healthy dietary patter as defined by Alternative Healthy Eating Index (AHEI-2010), dietary diversity or health diet scores.

### GRS and dietary components on BMI from observational studies

A total of 29 studies reported the interactions between GRS comprised of multiple SNPs and dietary components on obesity related phenotypes. These mainly investigated interactions with dietary fats (n = 11) (Alsulami, Nyakotey, et al. 2020; Casas-Agustench et al. 2014; Celis-Morales et al. 2017; Ding et al. 2018; Goni et al. 2015; Huang, Wang, Heianza, Zheng, et al. 2019; Lee et al. 2021; Lemas et al. 2013; Nakamura et al. 2016; Riedel et al. 2013; Rukh et al. 2013), or dietary patterns (n = 10) (Ding et al. 2018; Han et al. 2020; Hosseini-Esfahani, Koochakpoor, Daneshpour, Sedaghati-khayat, et al. 2017; Hosseini-Esfahani et al. 2019; Jaaskelainen et al. 2013; Masip et al. 2020; Nettleton et al. 2015; Seral-Cortes et al. 2020; Sotos-Prieto et al. 2020; Wang et al. 2018). While a smaller number of studies (n = 8) examined other dietary components including protein, calcium, Vitamin C, coffee and sweetened soft drinks intakes (Alathari et al. 2021; Rohde et al. 2017; Alsulami, Aji, et al. 2020; Ankarfeldt et al. 2014; Larsen et al. 2014b; Larsen et al. 2014a; Olsen et al. 2016; Wang et al. 2017)

**Figure 2.**
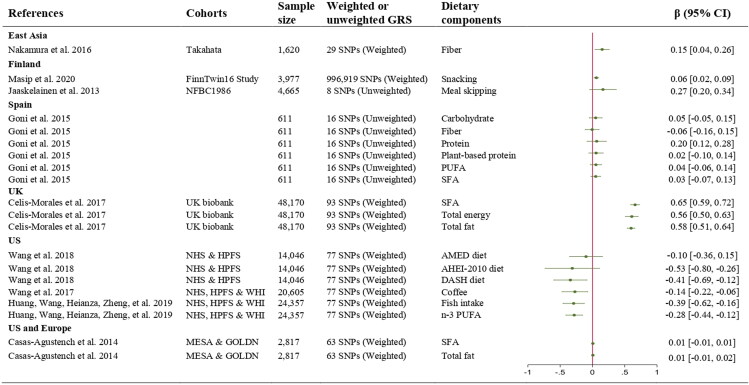
Forest plot of the interactions between GRS and dietary components on BMI (kg/m^2^) from observational studies. CI; confidence interval, GRS; genetic risk scores, PUFA; polyunsaturated fats, SFA; saturated fats, SNPs; single nucleotide polymorphisms

The number of SNPs included in the GRSs utilized in these studies ranged from 2 to 1,148,565 (Masip et al. 2020). A forest plot illustrates the GRS-diet interaction on BMI using β coefficient and 95% CI (Figure 2). Given the tremendous heterogeneity in the investigated dietary components, as well as the genetic variants included, and approaches taken to estimate the GRS the results are presented without calculating a summary estimate. In Europe, studies from Finnish populations, including FinnTwin16 (FT16) and Northern Finland Birth Cohort of 1986 (NFBC1986), reported that unhealthy dietary practices such as snacking (β = 0.27, 95% CI = 0.21, 0.34) (Masip et al. 2020) and meal skipping (β = 0.06, 95% CI = 0.02, 0.09) (Jaaskelainen et al. 2013) were associated with increased BMI among those with higher GRS based on 996,919 and 8 SNPs, respectively. Separately, findings from the UK Biobank reported that intakes of total energy (β = 0.56, 95% CI = 0.50, 0.63), total fat (β = 0.58, 95% CI = 0.51, 0.64), and SFA (β = 0.65, 95% CI = 0.59, 0.72) were associated with increased BMI among those with higher GRS based on 93 SNPs (Celis-Morales et al. 2017).

In the US, large cohort studies such as Health Professionals Follow-Up Study (HPFS), Nurses’ Health Study (NHS) and Women’s Health Initiative (WHI) demonstrated that increased adherence to AHEI-2010 (β=-0.53, 95% CI=-0.80, −0.26) and DASH diets (β=-0.41, 95% CI=-0.69, −0.12) (Wang et al. 2018). In addition, increased consumption of n-3 PUFA (β=-0.28, 95% CI=-0.44, −0.12), fish (β=-0.39, 95% CI=-0.62, −0.16) (Huang, Wang, Heianza, Zheng, et al. 2019) and coffee (β=-0.14, 95% CI=-0.22, −0.06) (Wang et al. 2017) were significantly associated with reduced BMI among those with higher GRS based on 77 SNPs. No significant interactions were found between GRS and intakes of total fat (β = 0.01, 95% CI=-0.01, 0.02) and SFA (β = 0.01, 95% CI=-0.01, 0.01) on BMI using the pooled data from the Multi-Ethnic Study of Atherosclerosis (MESA) and GOLDN cohorts comprising US and European populations (Casas-Agustench et al. 2014). While most studies were conducted in US and European populations, only limited data were found in the Asian populations.

Findings from an East Asian population who were genetically predisposed to obesity, the Takahata cohort study, showed that fiber intake was positively associated with BMI (β = 0.15, 95% CI = 0.04, 0.26); however, surprisingly per gram increases in vegetable fat and animal protein intake resulted in lower BMI (Nakamura et al. 2016). The authors opined that dietary fiber alone may not be sufficient to control weight among the participants with higher GRS, which may be due to the associated macronutrient intake that collectively affected the body weight. Furthermore, the authors note there are different types of fiber e.g., soluble or insoluble fiber, which were not differentiated in their analysis.

### Intervention trials: Effects of gene-diet interactions on weight loss outcomes

Studies (n = 56) that investigated genetic effects on weight loss outcomes in response to dietary interventions are summarized in Table 3. Low and very-low calorie diets (LCD and VLCD), and low-fat diets including high MUFA or PUFA and low SFA and cholesterol diets were the most investigated dietary interventions. Mixed results were found for the effects of genetic variants on weight loss outcomes in response to LCD or VLCD (n = 21 positive impact; n = 36 no impact; and n = 7 negative impact). In brief, calorie-restricted diets showed greater weight loss outcomes in individuals carrying minor alleles of SNPs in the: Amylase Alpha 1 A (*AMY1*) (US), Leptin (*LEP*) (French), Leptin Receptor (*LEPR*) (Spanish), Perilipin (*PLIN1-PLIN6*) (Belgium, but not in Spanish), Fatty Acid-Binding Protein 2 (*FABP2*), Superoxide Dismutase 2 (*SOD2)* (Mexican), ATP Binding Cassette Subfamily A Member 1 (*ABCA1*) (Brazilian), Uncoupling Protein 2 (*UCP2*) and *UCP3* (Korean), Peroxisome Proliferator-Activated Receptor Gamma (*PPARG*), and Adiponectin (*ADIPOQ*) (Japanese) genes (Abete et al. 2009; Cha et al. 2007; Heianza et al. 2017; Hernandez-Guerrero et al. 2018; Martinez-Lopez et al. 2013; Matsuo et al. 2009; Soenen et al. 2009; Teixeira et al. 2020; Tsuzaki et al. 2009; Yoon et al. 2007).

Whereas, unfavorable or no changes in weight loss outcomes in response to calorie-restricted diets were reported in individuals carrying the minor allele of SNPs in: Glutathione Peroxidase 1 (*GPX1*) and *SOD1* (Mexican), *PLIN1*, Brain-Derived Neurotrophic Factor (*BDNF*) and Glucagon-Like Peptide-1 Receptor (*GLP-1R)* (Spanish), *PPARG*, Fat Mass And Obesity-Associated (*FTO*), Angiotensin I-Converting Enzyme (*ACE*), *ADIPOQ* and Angiotensin II Type 2 Receptor (*AT2R*) (Japanese), ATP-Binding Cassette Super-Family G member 2 (*ABCG*) (Brazilian), *UCP2* and *UCP3* (Korean), and Sarcoglycan Gamma (*SGCG*) (Canadian) genes (Cha et al. 2007; de Luis et al. 2014; de Luis, Fernández Ovalle, et al. 2018; Hamada et al. 2011; Hernandez-Guerrero et al. 2018; Matsuo et al. 2009; Matsuo et al. 2012; Nikpay et al. 2020; Ruiz et al. 2011; Teixeira et al. 2020; Tsuzaki et al. 2009; Yoon et al. 2007).

Interestingly, the beneficial effects of low-fat diets (20-25% energy from fat) in reducing body weight and other obesity-related phenotypes including WC, fat mass and visceral fat were consistently reported in US, European, Spanish and Israel populations carrying the minor alleles of multiple SNPs including: Adrenoceptor Beta 3 (*ADRB3*) rs4994, Adenylate Cyclase 3 (*ADCY3*) rs10182181, HNF1 Homeobox A (*HNF1A*) rs7957197, Histamine N-methyltransferase (*HNMT*) rs12691940, Melatonin Receptor 1B (*MTNR1B*) rs10830963, Phosphofructokinase (*PFKL*) rs2838549, Retinoic Acid Receptor Beta (*RARB*) rs322695, Transcription Factor 7-Like 2 (*TCF7L2*) rs12255372, Transcription Factor AP-2 Beta (*TFAP2B*) rs987237, Fibroblast Growth Factor 21 (*FGF21*) rs838147, as well as individuals with higher GRS (e.g. computed from 5 to 96 SNPS) (Goni et al. 2019; Huang et al. 2018; Li et al. 2020; Mattei et al. 2012; Seip et al. 2008; Stocks et al. 2012). Whereas unfavorable effects on weight loss outcomes were found in the US and European populations carrying the minor alleles of Melatonin Receptor 1B (*MTNR1B*) rs10830963, *FGF21* rs838147, Vascular endothelial growth factor A (*VEGFA*) rs1358980, and individuals with higher GRS computed from 47 SNPs (Goni et al. 2019; Grau et al. 2010; Heianza et al. 2016; Svendstrup et al. 2018).

However, other studies reported low-carbohydrate-high fat diets (30-45% of energy from carbohydrate and 40-45% energy from fat) were more effective in reducing body weight and fat mass among those carrying the minor alleles of *HNF1A* rs7957197, Neuropeptide Y (*NPY*) rs16147, *TFAP2B* rs987237 in US and European populations (Huang et al. 2018; Lin et al. 2015; Stocks et al. 2012), but not among the risk alleles carriers of *FTO* rs9939609 (A), Angiotensin II Receptor Type 2 (*AGTR2*) rs5950584 (T) and *VEGFA* rs1358980 (T) (de Luis, Aller, Izaola, de la Fuente, et al. 2012; Seip et al. 2008; Svendstrup et al. 2018). This may be dependent on fat quality, as other studies reported that MUFA- and PUFA-enriched diets were found to be effective in reducing body weight and fat mass among the Spanish who carrying the risk alleles of Fatty Acid Amide Hydrolase (*FAAH*) rs32440 and Fatty Acid Binding Protein 2 (*FABP2*) Ala54Thr, respectively (de Luis, Aller, Izaola, Sagrado, et al. 2012; de Luis, Aller, Izaola, Conde, et al. 2013).

Findings from the US POUND LOST trial (n = 692; 61% females) showed that overweight and obese individuals with a lower GRS computed from 7 SNPs had significantly greater reduction in body weight (p = 0.003) and WC (p = 0.014) after 6 months of consuming low-fat diets compared to those with higher GRS (Li et al. 2020). On the other hand, the US MOVE! Programme (n = 51 overweight and obese adults; 25% females) reported no significant difference in weight loss outcomes after 24 weeks between those following nutrigenetic guided diets and standard balanced diet (Frankwich et al. 2015). In a European population, the NUGENOB trial reported that women with the highest decile of a GRS had significantly greater reduction in weight compared to the lowest decile (7.3 ± 3.0 kg versus 4.9 ± 3.0 kg) after 10 weeks of low-fat or low-carbohydrate hypocaloric diets (reduction of 600 kcal/day) (Svendstrup et al. 2018). Plus greater weight loss was observed in Dutch adults with higher GRS (β ± SE= −0.52 ± 0.18, p = 0.004) after consuming protein-enriched VLCD for 5 months (Verhoef et al. 2014). However, individuals with low GRS and high adherence to MD had a greater reduction in BMI and WC compared to low MD (San-Cristobal et al. 2017). However, in an Asian population, no significant effect of GRS was found on weight loss outcomes in response to both the HIPCREF (Individualized high-protein, energy-restricted, high-vitamin E and high-fiber) diet and a standard diet (based on Malaysian Dietary Guidelines 2010) among the overweight and obese Malaysian adults (Tan and Mitra 2020), but those with higher GRS had a significantly greater reduction in C-reactive protein levels after HIPCREF diet compared to the standard diet.

### Effects of gene-diet interactions on micronutrient status

Very few studies (5 observational studies and 4 intervention trials) investigated gene-diet interactions on micronutrient status (Table 4). From these, only Aldehyde Dehydrogenase 2 Family Member (*ALDH2*) rs671 was found to modulate both BMI and micronutrient status (Tao et al. 2019; Wang et al. 2016). Chinese men carrying A alleles of *ALDH2* rs671 who consumed alcohol (both > 0 < 10 g/day and ≥10 g/day groups) had significantly lower serum ferritin levels compared to men carrying GG genotypes (Tao et al. 2019), but this association was not observed in the non-alcohol drinkers. While in a separate study, Chinese men carrying A alleles of rs671 were observed to have lower visceral fat accumulation with lower alcohol consumption (OR = 0.27, CI = 0.09-0.23, per copy of A allele), suggesting a genetic interaction mediating BMI in the context of alcohol consumption (Wang et al. 2016).

**Table 4.** Human studies examining the effects of gene-diet interactions on micronutrient status.

Reference	Sample size & population	Age (years)	BMI (kg/m^2^)	SNPs	Dietary component or intervention	Main outcome	Risk of bias (general)^1^	Risk of bias (genetic)^2^
**Observational studies**								
Hiraoka 2004	340 Japanese women	20-22	NS	*MTHFR* C677T (C > T)	Vitamin B9 Folate (≥200µg/d)	Serum folate ↓	Low	Neutral
Cummings et al. 2017	265 US	17-34	NS	*FOLH1* T484C (T > C)	Vitamin B9 Folate (natural vs synthetic)	Red blood cell folate ↑	Low	Neutral
Cade et al. 2005 2005	2,528 UK women	35-69	24.5 ± 4.4	*HFE* H63D (H > D) and C282Y (C > Y)	Heme iron	Serum ferritin ↑	Low	Neutral
Tao et al. 2019	3,295 Chinese men	20-69	23.3 ± 3.4	*ALDH2* rs671 (A > G)	Alcohol	Serum folate ↑	Low	Low
Kokaze et al. 2014	436 Japanese men	54 ± 8	23.5 ± 2.6	Mitochondrial DNA (Mt) rs28357984 (C > A)	Coffee (≥4 cup/d)	Anemia risk ↑	Low	Neutral
**Intervention studies**								
Arias et al. 2017	34 Colombian women	25 ± 3	23.5 ± 3.6	*MTHFR* 677C→T, 1298A > C, and CbS 844ins68 (D > I)	400μg/day of folate supplementation for 3 months (pre-post study)	Serum folate ↔	Neutral	Low
Guinotte et al. 2003	43 Mexican women	18-45	19.5–32	*MTHFR* 677C→T	Folate repletion phase with 400 or 800 µg/d DFE of folate supplementation for 7 weeks	Serum folate ↓ urinary folate ↓	Neutral	Neutral
Solis et al. 2008 2008	60 Mexican American	18-55	<35	*MTHFR* 677C→T	438μg DFE/d (RDA) and total choline intakes of 300, 550, 1100, or 2200 mg/d for 12 weeks (pre-post study)	Serum folate reduced ↓ tHcy ↑	Neutral	Neutral
Lisboa et al. 2020	48 Brazilian women	45	30	*MTHFR* 677C→T	Group 1 received 95μg/d folate and 300g vegetables and Group 2 received 191μg/d folate and 300g vegetables for 8 weeks	Serum folate ↔	Low	Neutral

Risk of bias (general) ^1^was assessed by Quality Criteria Checklist and risk of bias (genetic) ^2^was assessed by a methodology quality evaluation method specifically tailored to gene-diet interaction research.

↓ indicates significant lower or greater reduction among the risk alleles carriers; ↑ indicates significant lower or smaller reduction among the risk alleles carriers; ↔ indicates no significant differences or post-intervention changes between the risk alleles and non-risk alleles carriers.

*ALDH2*; Aldehyde Dehydrogenase 2 Family Member, BMI; body mass index, DFE; Dietary Folate Equivalent, *FOLH1*; Folate Hydrolase 1, *HFE*; Homeostatic Iron Regulator, *MTHFR*; Methylene tetrahydrofolate reductase; NS, not stated; RDA; Recommended Daily Allowance, tHcy; total homocysteine.

Higher red blood cell folate levels were observed with increased folate intake in the US population carrying the TT genotype of Folate Hydrolase 1 (*FOLH1*) T484C, compared to C allele carriers (Cummings et al. 2017). However, Japanese women carrying the TT genotype of Methylene tetrahydrofolate reductase (*MTHFR*) 677 C > T had significantly lower serum folate with increased intake of folic acid compared to non-carriers (Hiraoka 2004). With respect to the intervention studies, four trials have investigated the modulatory effect of *MTHFR* 677 C > T on serum or urinary folate levels in response to folate supplementation (95, 191, 400 or 800 μg/d of folate). No significant post-intervention (8 weeks to 3 months) differences were observed in serum folate among Colombian and Brazilian women (Arias et al. 2017; Lisboa et al. 2020). However, in two separate studies of Mexican women and men, the T alleles carriers of MTHFR 677 C > T had significantly lower serum folate levels after folate supplementation (12-14 weeks) compared to non-carriers (Guinotte et al. 2003; Solis et al. 2008).

Separately, UK women carrying the YY genotype of the Homeostatic Iron Regulator (*HFE*) C282Y allele had significantly higher serum ferritin with increased heme iron intake (Cade et al. 2005). Increased risk of anemia with higher consumption of coffee (≥4 cup/d) was reported in those carrying the C allele of Mitochondrial DNA rs28357984 (Kokaze et al. 2014).

## Discussion

In this study we comprehensively reviewed the current literature on the effects of gene-diet interactions on nutritional status. This included examining the effect of genetic variants on undernutrition, overnutrition and micronutrient status (iron, zinc, folate and vitamin A), and in response to nutrient or dietary intakes.

### GRS and dietary patterns on obesity

The vast majority of the identified studies focused on the impacts of gene-diet interactions on BMI or obesity risk, while only a limited number reported data related to under-nutrition and micronutrient status. Notably, most of the interaction findings have yet to be replicated in controlled trials or across diverse populations, with a particular paucity of data from Asian populations. Both observational studies and intervention trials consistently demonstrated the beneficial effects of avoiding diets that are high in total fat, SFA, TFA and n-6 PUFA; as well as increasing MUFA and n-3 PUFA intakes, for reducing obesity risk and obesity-related phenotypes and providing better weight loss outcomes among individuals genetically predisposed to obesity.

Most of the observational studies to date have focused on the investigation of individual nutrients, food items, or individual SNPs. However, people do not consume single nutrients or foods but rather a combination of many foods, and the complexity and multidimensionality of a normal diet can confound dietary intervention studies. To address this issue, analysis of food consumption patterns can be measured using both *a priori* and *a posteriori* approaches (Hu 2002). Prospective approaches measure either an individual’s adherence to a specific diet e.g., Mediterranean, AMED, or DASH diets; or measure diet quality through scores e.g., AHEI-2010. For retrospective approaches, these typically use principal components analysis (PCA) and cluster analyses to assess diet patterns based on foods consumed, such as the Western, plant-based or meat-based diets (Stricker et al. 2013).

Genetic variants most commonly investigated in gene-diet interaction studies in relation to obesity, were SNPs in the *FTO* gene (rs9939609, rs1121980 and rs1421085), followed by *MC4R* (rs1778231), *PPARG* (rs1801282) and *APOA5* (rs662799) genes (Livingstone, Celis-Morales, Papandonatos, et al. 2016; Razquin, Marti, and Martinez 2011; Tan, Mitra, and Amini 2018; Xiang et al. 2016). There were consistent evidence showing the associations between increased intake of total fat and SFA and increased BMI in the individuals carrying the risk allele of *FTO* rs9939609, rs1121980 and rs1421085, including those assessed by GRS with *FTO* SNPs inclusive (Alsulami, Nyakotey, et al. 2020; Celis-Morales et al. 2017; Corella et al. 2007; Czajkowski et al. 2020; Goni et al. 2015; Sonestedt et al. 2009; Sonestedt et al. 2011; Labayen et al. 2016; Park et al. 2013), and such associations were not observed in other macronutrients. Whereas increased intake of PUFA (Goni et al. 2015; Huang, Wang, Heianza, Wiggs, et al. 2019; Lemas et al. 2013; Riedel et al. 2013) or increased adherence to diets rich in vegetables, fruits and whole grains that assessed using the scoring systems such as MD, DASH, AMED, AHEI-2010, dietary diversity and healthy diet index scores (Ding et al. 2018; Goodarzi et al. 2021; Han et al. 2020; Seral-Cortes et al. 2020; Wang et al. 2018) showed better reduction in BMI and body fatness in the *FTO* risk allele carriers compared to the non-carriers.

Both *FTO* and *MC4R* genes are involved in the regulation of appetite and energy intake (Adan et al. 2006; Fawcett and Barroso 2010; Olszewski et al. 2009), while *PPARG* and *APOA5* mediate adaptive thermogenesis (Wu, Cohen, and Spiegelman 2013) and lipoprotein metabolism (Su, Kong, and Peng 2018) respectively. Nonetheless, inconclusive findings were found on the interactions between dietary intake and the SNPs in *MC4R*, *PPARG* and *APOA5* genes. However, it is established that obesity is a polygenic trait, and an individual’s susceptibility to obesity is a result of the combined effect of many variants in many genes (Loos 2009). Researchers are addressing this through the analyses of GRS or polygenic risk scores (PRS), which are computed as a weighted sum of trait associated-risk alleles (Lewis and Vassos 2020).

Early work examining the hereditary basis of height demonstrated that GRS models that include large number of SNPs, each with effects too small to be detected individually, may better explain the molecular basis of complex traits and diseases, than using a smaller number of SNPs with confirmed associations (Yang et al. 2010). It is hoped that ultimately, GRS may both explain the variation observed between the populations and lead to improved disease prevention and treatment. In the studies examined within this review, the number of SNPs included in computed GRS for obesity ranged from 2 to 1,148,565 SNPs. This heterogeneity highlights the current challenge of how to utilize the limited data from genetic-association studies that identify individual SNPs with significant functional effects, while also determining the optimum number of SNPs to be included in the computation of GRS (Crouch and Bodmer 2020).

A consistent finding was for the beneficial effects of increased adherence to high-quality diets such as MD, AMED, and DASH diets in reducing obesity risk among individuals who had high GRS compared to those with low GRS (Ding et al. 2018; Han et al. 2020; Hosseini-Esfahani, Koochakpoor, Daneshpour, Sedaghati-khayat, et al. 2017; Seral-Cortes et al. 2020; Sotos-Prieto et al. 2020; Wang et al. 2018). Unfavorable effects of both Western diets and meal skipping on obesity indicators were also observed among those had high GRS compared to low GRS (Hosseini-Esfahani et al. 2019; Jaaskelainen et al. 2013; Masip et al. 2020; Nettleton et al. 2015). Individuals carrying higher GRS that had an increased adherence to MD were observed to have 0.67 times lower risk of obesity in Iranian populations (Hosseini-Esfahani, Koochakpoor, Daneshpour, Sedaghati-khayat, et al. 2017); as well as decreased adiposity in European and US populations (mean difference ± SD in BMI: −1.5 ± 0.67 kg/m^2^) (Seral-Cortes et al. 2020; Sotos-Prieto et al. 2020).

In addition, increased adherence to AMED and DASH diets, or higher scores in the AHEI-2010, in individuals with higher GRS were also associated with reduced BMI in the US and European populations (Ding et al. 2018; Wang et al. 2018). Therefore, the results promisingly suggest for individuals with high GRS an even greater beneficial impact on reducing risk of obesity from the avoidance of dietary patterns high in red/processed meats and sugary, fried or fatty foods, and inclusion of a variety of vegetables, fruits and cereals in the diet. However, these findings were mainly obtained from observational studies and intervention trials are warranted to confirm these findings.

### Limited research on micronutrient status and under-nutrition

Although the genetic susceptibility to obesity and its interactions with dietary components have been extensively investigated, there are scarce data on the influence of gene-diet interactions on thinness or under-nutrition and micronutrient status. With respect to blood micronutrient status, our review finds that MTHFR 677 C > T were the most common SNPs associated with serum folate levels, with individuals carrying the T allele had significantly lower serum folate levels compared to the non-carriers after intervening with folate supplementation or diets high in folate intake among the Mexican (Guinotte et al. 2003; Solis et al. 2008) and Japanese women (Hiraoka 2004), although this effect was not observed in Colombia (Arias et al. 2017) and Brazilian women (Lisboa et al. 2020). On contrary, observational studies reported that polymorphism in *ALDH2* rs671 (Tao et al. 2019) was associated with significantly higher folate levels with higher intake of alcohol, whereas *HFE* C282Y (Cade et al. 2005) and *FOLH1* T484C (Cummings et al. 2017) were associated significantly higher ferritin levels with higher intakes of heme iron and folate, respectively.

In this review, we only found one study reported the interaction between *IGF* rs680 and dairy products on body height in the children (Dedoussis et al. 2010). However, this is understandable as the low prevalence of extreme thinness may have posed challenges to the recruitment of healthy thin individuals to study the effects of gene-diet interactions on undernutrition, and hence contributes to the data scarcity. A recent study suggested that thinness is, like obesity, a heritable trait. The authors identified 10 loci, previously found to be associated with obesity were also influencing thinness. These SNPs included *FTO* rs9930333, *MC4R* rs2168711 and Transmembrane Protein 18 (*TMEM18*) rs6748821 (Riveros-McKay et al. 2019). In a separate study, Apolipoprotein H (*APOH*) rs52797880 was reported as an obesity-resistance gene that interacted with *FTO* rs9939609 and doubled the odds of thinness (Hasstedt et al. 2016). It has been proposed that the inheritance of thinness may exert mild protective effect in mitigating against the development of obesity caused by the environmental factors such as dietary components and physical activity levels (Costanzo and Schiffman 1989), which deserves further investigation.

### Quality of available gene-diet interaction studies

An adequate sample size is a critical component in gene-diet interaction studies to avoid underpowered statistical analyses (Gauderman 2002). Low statistical power reduces the chance of detecting a true interaction and may produce false negative findings. It has been evidenced that a minimum sample size of ∼6500 is needed in a case-control study design to achieve a 80% power to detect a gene-diet interaction (with an OR of ∼1.5) with a 50% allele frequency in the population (García-Closas and Lubin 1999). Based on the studies included in this review, the 15^th^ and 75^th^ percentile of the total sample size of the intervention studies ranged from 75 and 365, respectively (Table S2), and almost 75% of the case-control studies had a sample size < 5000.

Sample size issues may be further exaggerated in studies investigating multiple genetic variants for interactions with dietary components, which require adjustment for multiple testing to avoid false positive findings (Bouaziz, Jeanmougin, and Guedj 2012). In fact, if the p values for the significance of the gene-diet interactions were to be adjusted for all the tested genetic variants, such interactions are unlikely to remain significant. Therefore, computation of GRS which combines all the SNPs tested may be able to address this issue by avoiding power loss due to the multiple-testing correction (Lin et al. 2019). Other factors such as adjustment for multiple ethnicities or populations, calculation for allele frequencies using Hardy-Weinberg Equilibrium, genetic model used, genotype relative risk (effect size) genotyping errors, accuracy of the measurements of exposures and outcomes may affect the sample size and limit the statistical power to evaluate gene-diet interaction (Gordon and Finch 2005).

### Strengths and limitations

There were some limitations to this current review. Studies varied dramatically in terms of their reported dietary components, dietary assessment methods and genetic variants. The high heterogeneity of the studies done to date increases the complexity of interpretation. Moreover, the majority of gene-diet interaction findings have not been replicated in multiple studies and were mainly reported from the US and European populations. Therefore, their findings may not be generalizable to other ethnicities or populations. In this review, only the interactions between genetic variations and dietary factors were evaluated, although we know that other factors such as physical activity, epigenetic and gut microbiome play a critical role in modulating nutritional status. These parameters are important determinants of obesity and potential confounding factors that should not be ignored.

Nonetheless, our study had some notable strengths. In particular, both observational studies and intervention trials were included to comprehensively examine the effects of gene-diet interactions on under and overnutrition and micronutrient deficiency. In addition, quality evaluation was conducted rigorously whereby we utilized an additional methodology quality evaluation tool specifically tailored to gene-diet interaction research. The majority of included studies were assessed as having either low or medium risk of bias. As the evaluation of dietary patterns and the combined effect of multiple genetic variants using GRS may provide better understanding on the complex gene-diet interaction, this thorough synthesis of the literature to date provides a useful tool for future research design.

## Conclusion

This systematic review reveals that most of the gene-diet interaction studies to date have focused on overnutrition. The findings suggest that healthy dietary patterns, characterized by the high intakes of whole grains, vegetables and fruits, and low intakes of total fat and SFA, may benefit individuals who had higher GRS compared to lower GRS, particularly those carrying the risk alleles of *FTO* SNPs (rs9939609, rs1121980 and rs1421085) in reducing or managing their body weight. Other SNPs in *MC4R*, *PPARG* and *APOA5* were also commonly studied for interactions with nutrients or diet in overnutrition though findings were inconclusive. However, most of the interaction findings identified to date have yet to be replicated in trials across multiple populations, more data from Asian populations are warranted. Notably, there are insufficient data available for drawing conclusions about gen-diet interactions and effects on undernutrition and micronutrient status. Although *MTHFR* 677 C > T was commonly found to be associated with serum folate levels, inconsistent findings were observed across different populations. Future gene-diet interaction research should focus on the investigation of both under and overnutrition, to better identify the most effective dietary patterns for personalized nutrition strategies to improve the human health.

## Supplementary Material

Supplemental Material
