# Supplementary material for: In the context of the triple burden of malnutrition: A systematic review of gene-diet interactions and nutritional status
Source: Crit Rev Food Sci Nutr. 2022 Oct 12;64(11):3235–63. doi: 10.1080/10408398.2022.2131727 (PMC11000749; doi:10.1080/10408398.2022.2131727)
Supplement: Supplemental Material [file BFSN_A_2131727_SM9779.zip › Supp/Revised Supplementary Table 3[AU].docx]

**Table S3: Outcomes of the risk of bias assessment for each of the study included (n=167)**

| **Author (years)** | **Methodological quality assessment for gene-diet interaction research** | | | | | | | | | **Quality Criteria Checklist** | | | | | | | | | | |
| --- | --- | --- | --- | --- | --- | --- | --- | --- | --- | --- | --- | --- | --- | --- | --- | --- | --- | --- | --- | --- |
|  | **Interaction as primary study goal** | **Statistical test for interaction** | **Correction for multiple testing** | **Correction for ethnicity** | **Hardy-Weinberg Equilibrium** | **Group similarity at baseline tested** | **Sample size/ power analysis** | **Sufficient study details** | **Score (full=8)** | **Research Question** | **No selection bias** | **Randomisation / group comparability** | **Withdrawal description** | **Blinding** | **Intervention/ exposure clearly described** | **Validity and realiability of outcome measures** | **Statistical analysis** | **Conclusion supported by results** | **No funding bias** | **Overall** |
| **Observational studies (n=107)** | | | | | | | | | | | | | | | | | | | | |
| Alathari et al. 2021 | 1 | 1 | 1 | 1 | 1 | 1 | -1 | 1 | 6 | Yes | Yes | Yes | Yes | Yes | Yes | Yes | Yes | Yes | Yes | Low |
| Alsulami, Aji, et al. 2020 | 1 | 1 | 1 | 1 | 1 | 1 | -1 | 1 | 6 | Yes | Yes | Yes | Yes | Yes | Yes | Yes | Yes | Yes | Yes | Low |
| Alsulami, Nyakotey, et al. 2020 | 1 | 1 | 1 | 1 | 1 | 1 | -1 | 1 | 6 | Yes | Yes | Yes | Yes | Yes | Yes | Yes | Yes | Yes | Yes | Low |
| Ankarfeldt et al. 2014 | 1 | 1 | 1 | 1 | -1 | 1 | 1 | 1 | 6 | Yes | Yes | Yes | Yes | Yes | Yes | Yes | Yes | Yes | Yes | Low |
| Barchitta et al. 2014 | 1 | 1 | 1 | 1 | 1 | 1 | -1 | 1 | 6 | Yes | Yes | Yes | Yes | Yes | Yes | Yes | No | Yes | Yes | Low |
| Bauman-fortin et al. 2019 | 1 | 1 | 1 | 1 | 1 | 1 | -1 | 1 | 6 | Yes | Yes | Yes | Yes | Yes | Yes | Yes | Yes | Yes | Yes | Low |
| Cade et al. 2015 | 1 | 1 | 1 | 0 | -1 | 1 | 0 | 1 | 4 | Yes | Yes | Yes | Yes | Yes | Yes | Yes | Yes | Yes | Yes | Low |
| Casas-Agustench et al. 2014 | 1 | 1 | 1 | 1 | 1 | 1 | 0 | 1 | 7 | Yes | Yes | Yes | Yes | Yes | Yes | Yes | Yes | Yes | Yes | Low |
| Celis-Morales et al. 2017 | 1 | 1 | 1 | 1 | 1 | 1 | 1 | 1 | 8 | Yes | Yes | Yes | Yes | Yes | Yes | Yes | Yes | Yes | Yes | Low |
| Chen et al. 2019 | 1 | 1 | 1 | 1 | 1 | 1 | 1 | 1 | 8 | Yes | Yes | Yes | Yes | Yes | Yes | Yes | Yes | Yes | Yes | Low |
| Corella et al. 2007 | 1 | 1 | -1 | 1 | 1 | 1 | 0 | 1 | 5 | Yes | Yes | Yes | Yes | Yes | Yes | Yes | Yes | Yes | Yes | Low |
| Corella et al. 2009 | 1 | 1 | 1 | 1 | 1 | 1 | 0 | 1 | 7 | Yes | Yes | Yes | Yes | Yes | Yes | Yes | Yes | Yes | Yes | Low |
| Corella et al. 2011 | 1 | 1 | 1 | 1 | -1 | 1 | 1 | 1 | 7 | Yes | Yes | Yes | Yes | Yes | Yes | Yes | Yes | Yes | Yes | Low |
| Cummings et al. 2017 | 1 | 1 | 1 | 0 | -1 | 1 | -1 | 1 | 3 | Yes | Yes | Yes | No | No | Yes | Yes | Yes | Yes | Yes | Low |
| Czajkowski et al. 2020 | 1 | 1 | -1 | 1 | 1 | 1 | -1 | 1 | 4 | Yes | Yes | Yes | Yes | Yes | Yes | Yes | Yes | Yes | Yes | Low |
| Davis et al. 2010 | 1 | 1 | 1 | 1 | 1 | 1 | -1 | 1 | 6 | Yes | Yes | Yes | Yes | No | Yes | Yes | Yes | Yes | Yes | Low |
| Dedoussis et al. 2010 | 1 | 1 | 1 | 1 | 1 | 1 | -1 | 1 | 6 | Yes | Yes | Yes | Yes | Yes | Yes | Yes | Yes | Yes | Yes | Low |
| Dedoussis et al. 2011 | 1 | 1 | 1 | 1 | 1 | 1 | 1 | 1 | 8 | Yes | Yes | Yes | Yes | Yes | Yes | Yes | Yes | Yes | Yes | Low |
| Ding et al. 2018 | 1 | 1 | 1 | 1 | 1 | 1 | 1 | 1 | 8 | Yes | Yes | Yes | Yes | Yes | Yes | Yes | Yes | Yes | Yes | Low |
| Domínguez-Reyes et al. 2015 | 1 | 1 | -1 | 1 | 1 | 1 | -1 | 1 | 4 | Yes | Yes | Yes | Yes | Yes | Yes | Yes | Yes | Yes | Yes | Low |
| Doo et al. 2014 | 1 | 1 | 1 | 1 | 1 | 1 | 1 | 1 | 8 | Yes | Yes | Yes | Yes | Yes | Yes | Yes | Yes | Yes | Yes | Low |
| Doo and Kim 2010 | 1 | 1 | 1 | 1 | 1 | 1 | 0 | 1 | 7 | Yes | Yes | Yes | Yes | Yes | Yes | Yes | Yes | Yes | Yes | Low |
| Dumont et al. 2018 | 1 | 1 | 1 | 1 | 1 | 1 | 0 | 1 | 7 | Yes | Yes | Yes | Yes | Yes | Yes | Yes | Yes | Yes | Yes | Low |
| Galmes et al. 2020 | 1 | 1 | 1 | 0 | -1 | 1 | -1 | 1 | 3 | Yes | Yes | Yes | Yes | No | Yes | Yes | Yes | Yes | Yes | Low |
| Garaulet et al. 2014 | 1 | 1 | 1 | 1 | -1 | 1 | 0 | 1 | 5 | Yes | Yes | Yes | Yes | Yes | Yes | Yes | Yes | Yes | Yes | Low |
| Garske et al. 2019 | 1 | 1 | 1 | 1 | -1 | 1 | 1 | 1 | 6 | Yes | Yes | Yes | Yes | Yes | Yes | Yes | Yes | Yes | Yes | Low |
| Gong et al. 2021 | 1 | 1 | -1 | 1 | 1 | 1 | 0 | 1 | 5 | Yes | Yes | Yes | Yes | Yes | Yes | Yes | Yes | Yes | Yes | Low |
| Goni et al. 2015 | 1 | 1 | 1 | 1 | 1 | 1 | -1 | 1 | 6 | Yes | Yes | Yes | Yes | No | Yes | Yes | Yes | Yes | Yes | Low |
| Goodarzi et al. 2021 | 1 | 1 | -1 | 1 | 1 | 1 | 0 | 1 | 5 | Yes | Yes | Yes | Yes | Yes | Yes | Yes | Yes | Yes | Yes | Low |
| Goodwin et al. 2015 | 1 | 1 | 1 | 1 | 1 | 1 | -1 | 1 | 6 | Yes | Yes | Yes | Yes | No | Yes | Yes | Yes | Yes | Yes | Low |
| Han et al. 2020 | 1 | 1 | 1 | 1 | -1 | 1 | 1 | 1 | 6 | Yes | Yes | Yes | Yes | Yes | Yes | Yes | Yes | Yes | Yes | Low |
| Higashibata et al. 2016 | 1 | 1 | 1 | 1 | 1 | 1 | 0 | 1 | 7 | Yes | Yes | Yes | Yes | Yes | Yes | Yes | Yes | Yes | Yes | Low |
| Hiraoka 2004 | 1 | 1 | 1 | 1 | -1 | 1 | -1 | 1 | 4 | Yes | Yes | Yes | Yes | No | Yes | Yes | Yes | Yes | Yes | Low |
| Hiroi et al. 2011 | 1 | 1 | 1 | 1 | -1 | 1 | 0 | 1 | 5 | Yes | Yes | Yes | Yes | Yes | Yes | Yes | Yes | Yes | Yes | Low |
| Hosseini-Esfabani et al. 2019 | 1 | 1 | 1 | 1 | 1 | 1 | 0 | 1 | 7 | Yes | Yes | Yes | Yes | Yes | Yes | Yes | Yes | Yes | Yes | Low |
| Hosseini-Esfahani et al. 2017 | 1 | 1 | 1 | 1 | 1 | 1 | 0 | 1 | 7 | Yes | Yes | Yes | Yes | Yes | Yes | Yes | Yes | Yes | Yes | Low |
| Huang et al. 2015 | 1 | 1 | 1 | 1 | 1 | 1 | 1 | 1 | 8 | Yes | Yes | Yes | Yes | Yes | Yes | Yes | Yes | Yes | Yes | Low |
| Huang, Wang, Heianza, Zheng, et al. 2019 | 1 | 1 | 1 | 1 | -1 | 1 | 1 | 1 | 6 | Yes | Yes | Yes | Yes | Yes | Yes | Yes | Yes | Yes | Yes | Low |
| Huang, Wang, Heianza, Wiggs, et al. 2019 | 1 | 1 | 1 | 1 | 1 | 1 | 1 | 1 | 8 | Yes | Yes | Yes | Yes | Yes | Yes | Yes | Yes | Yes | Yes | Low |
| Huriyati et al. 2016 | 1 | -1 | 1 | 1 | -1 | 1 | -1 | 1 | 2 | Yes | Yes | Yes | Yes | No | Yes | No | No | Yes | Yes | Neutral |
| Huriyati et al. 2020 | 1 | 1 | 1 | 1 | -1 | 1 | -1 | 1 | 4 | Yes | Yes | Yes | Yes | No | Yes | Yes | Yes | Yes | Yes | Low |
| Jaaskelainen et al. 2013 | 1 | 0 | 1 | 1 | 1 | 1 | 0 | 1 | 6 | Yes | Yes | Yes | Yes | Yes | Yes | Yes | No | Yes | Yes | Low |
| Jiang et al. 2019 | 1 | 1 | 1 | 1 | 1 | 1 | 0 | 1 | 7 | Yes | Yes | Yes | Yes | Yes | Yes | No | Yes | Yes | Yes | Neutral |
| Joffe et al. 2014 | 1 | 1 | 1 | 1 | 1 | 1 | -1 | 1 | 6 | Yes | Yes | Yes | Yes | Yes | Yes | Yes | Yes | Yes | Yes | Low |
| Junyent et al. 2010 | 1 | 1 | -1 | 1 | 1 | 1 | -1 | 1 | 4 | Yes | Yes | Yes | Yes | Yes | Yes | Yes | Yes | Yes | Yes | Low |
| Kokaze et al. 2014 | 1 | 1 | 1 | 1 | -1 | 1 | -1 | 1 | 4 | Yes | Yes | Yes | Yes | No | Yes | Yes | Yes | Yes | Yes | Low |
| Labayen et al. 2016 | 1 | 1 | 1 | -1 | 1 | 1 | -1 | 1 | 4 | Yes | Yes | Yes | Yes | Yes | Yes | Yes | Yes | Yes | Yes | Low |
| Larsen et al. 2014 | 1 | 1 | 1 | 1 | -1 | 1 | 1 | 1 | 6 | Yes | Yes | Yes | Yes | Yes | Yes | Yes | Yes | Yes | Yes | Low |
| Larsen et al. 2014 | 1 | 1 | 1 | 1 | -1 | 1 | 1 | 1 | 6 | Yes | Yes | Yes | Yes | Yes | Yes | Yes | Yes | Yes | Yes | Low |
| Latella et al. 2009 | 1 | 1 | 1 | 1 | 1 | 1 | -1 | 1 | 6 | Yes | Yes | Yes | Yes | Yes | Yes | Yes | Yes | Yes | Yes | Low |
| Lee et al. 2017 | 1 | 1 | 1 | 1 | 1 | 1 | -1 | 1 | 6 | Yes | Yes | Yes | Yes | No | Yes | Yes | Yes | Yes | Yes | Low |
| Lee et al. 2021 | 1 | 1 | 1 | 1 | 1 | 1 | 1 | 1 | 8 | Yes | Yes | Yes | Yes | Yes | Yes | Yes | Yes | Yes | Yes | Low |
| Lemas et al. 2012 | 1 | 1 | 1 | 1 | 1 | 1 | 0 | 1 | 7 | Yes | Yes | Yes | Yes | Yes | Yes | Yes | Yes | Yes | Yes | Low |
| Li et al. 2019 | 1 | 1 | 1 | 0 | 1 | 1 | 1 | 1 | 7 | Yes | Yes | Yes | Yes | Yes | Yes | Yes | Yes | Yes | Yes | Low |
| Lim et al. 2014 | 1 | 1 | 1 | 1 | 1 | 1 | 0 | 1 | 7 | Yes | Yes | Yes | Yes | Yes | Yes | Yes | Yes | Yes | Yes | Low |
| Livingstone et al. 2016 | 1 | 1 | 1 | -1 | 1 | 1 | 0 | 1 | 5 | Yes | Yes | Yes | No | Yes | Yes | Yes | Yes | Yes | Yes | Low |
| Lv et al. 2015 | 1 | 1 | 1 | 1 | 1 | 1 | 0 | 1 | 7 | Yes | Yes | Yes | Yes | Yes | Yes | Yes | Yes | Yes | Yes | Low |
| Ma et al. 2014 | 1 | 1 | 1 | 1 | 1 | 1 | 1 | 1 | 8 | Yes | Yes | Yes | Yes | Yes | Yes | Yes | Yes | Yes | Yes | Low |
| Mansego et al. 2015 | 1 | 1 | 1 | 1 | 1 | 1 | -1 | 1 | 6 | Yes | Yes | Yes | Yes | Yes | Yes | Yes | Yes | Yes | Yes | Low |
| Marcos-pasero et al. 2019 | 1 | 1 | 1 | 1 | -1 | 1 | -1 | 1 | 4 | Yes | Yes | Yes | Yes | Yes | Yes | Yes | Yes | Yes | Yes | Low |
| Martinez et al. 2003 | 1 | 1 | 1 | 1 | 1 | 1 | -1 | 1 | 6 | Yes | Yes | Yes | Yes | No | Yes | Yes | Yes | Yes | Yes | Low |
| Masip et al. 2020 | 1 | 1 | 1 | 1 | 1 | 1 | 0 | 1 | 7 | Yes | Yes | Yes | Yes | Yes | Yes | Yes | Yes | Yes | Yes | Low |
| Merritt et al. 2018 | 1 | 1 | 1 | 1 | -1 | 1 | 0 | 1 | 5 | Yes | Yes | Yes | Yes | Yes | Yes | Yes | Yes | Yes | Yes | Low |
| Miyaki et al. 2005 | 1 | 1 | 1 | 1 | 1 | 1 | -1 | 1 | 6 | Yes | Yes | Yes | Yes | No | Yes | Yes | Yes | Yes | Yes | Low |
| Mollahosseini et al. 2020 | 1 | 1 | 1 | 1 | 1 | 1 | -1 | 1 | 6 | Yes | Yes | Yes | Yes | Yes | Yes | Yes | No | Yes | Yes | Low |
| Mook-Kanamori et al. 2009 | 1 | 1 | 1 | -1 | 1 | 1 | 0 | 1 | 5 | Yes | Yes | Yes | No | Yes | Yes | Yes | Yes | Yes | Yes | Low |
| Mousavizadeh et al. 2020 | 1 | 1 | -1 | 1 | 1 | 1 | 0 | 1 | 5 | Yes | Yes | Yes | Yes | Yes | Yes | Yes | Yes | Yes | Yes | Low |
| Muhammad et al. 2019 | 1 | 1 | 1 | 1 | -1 | 1 | -1 | 1 | 4 | Yes | Yes | Yes | Yes | No | Yes | Yes | Yes | Yes | Yes | Low |
| Nakamura et al. 2016 | 1 | 1 | 1 | 1 | 1 | 1 | 0 | 1 | 7 | Yes | Yes | Yes | Yes | Yes | Yes | No | Yes | Yes | Yes | Neutral |
| Nasreddine et al. 2019 | 1 | 1 | 1 | 1 | 1 | 1 | -1 | 1 | 7 | Yes | Yes | Yes | Yes | Yes | Yes | No | Yes | Yes | Yes | Low |
| Nettleton et al. 2015 | 1 | 1 | 1 | 1 | 1 | 1 | 1 | 1 | 8 | Yes | Yes | Yes | Yes | Yes | Yes | Yes | Yes | Yes | Yes | Low |
| Nieters et al. 2002 | 1 | 1 | -1 | 1 | 1 | 1 | -1 | 1 | 4 | Yes | Yes | Yes | Yes | Yes | Yes | Yes | Yes | Yes | Yes | Low |
| Olsen et al. 2016 | 1 | 1 | 1 | 1 | -1 | 1 | 0 | 1 | 5 | Yes | Yes | Yes | Yes | Yes | Yes | Yes | Yes | Yes | Yes | Low |
| Park et al. 2013 | 1 | 1 | 1 | 1 | 1 | 1 | 1 | 1 | 8 | Yes | Yes | Yes | Yes | Yes | Yes | Yes | Yes | Yes | Yes | Low |
| Park et al. 2016 | 1 | 1 | 1 | 1 | 1 | 1 | 1 | 1 | 8 | Yes | Yes | Yes | Yes | Yes | Yes | Yes | Yes | Yes | Yes | Low |
| Riedel et al. 2013 | 1 | 1 | 1 | 1 | -1 | 1 | 0 | 1 | 5 | Yes | Yes | Yes | Yes | Yes | Yes | Yes | Yes | Yes | Yes | Low |
| Robitaille et al. 2003 | 1 | 1 | 1 | 1 | -1 | 1 | -1 | 1 | 4 | Yes | Yes | Yes | No | Yes | Yes | Yes | Yes | Yes | Yes | Low |
| Robitaille, Houde, et al. 2007 | 1 | 1 | 1 | 1 | 1 | -1 | -1 | 1 | 4 | Yes | Yes | No | No | Yes | Yes | No | No | Yes | Yes | Neutral |
| Robitaille, Perusse, et al. 2007 | 1 | 1 | -1 | 1 | 1 | 1 | -1 | 1 | 4 | Yes | Yes | Yes | No | Yes | Yes | No | Yes | Yes | Yes | Neutral |
| Rocha et al. 2018 | 1 | 1 | 1 | 1 | 1 | 1 | 0 | 1 | 7 | Yes | Yes | Yes | Yes | Yes | Yes | Yes | Yes | Yes | Yes | Low |
| Rohde et al. 2017 | 1 | 1 | 1 | 1 | 1 | 1 | 1 | 1 | 8 | Yes | Yes | Yes | Yes | Yes | Yes | Yes | Yes | Yes | Yes | Low |
| Rukh et al. 2013 | 1 | 1 | 1 | 1 | 1 | 1 | 1 | 1 | 8 | Yes | Yes | Yes | Yes | Yes | Yes | Yes | Yes | Yes | Yes | Low |
| Rukh et al. 2017 | 1 | 1 | 1 | 1 | 1 | 1 | 0 | 1 | 7 | Yes | Yes | Yes | Yes | Yes | Yes | Yes | Yes | Yes | Yes | Low |
| Sanchez-Moreno et al. 2010 | 1 | 1 | 1 | 1 | -1 | 1 | 0 | 1 | 5 | Yes | Yes | Yes | Yes | No | Yes | No | Yes | Yes | Yes | Neutral |
| Seral Cortes 2020 | 1 | 1 | 1 | -1 | 1 | 1 | -1 | 1 | 4 | Yes | Yes | Yes | Yes | Yes | Yes | Yes | Yes | Yes | Yes | Low |
| Smith et al. 2008 | 1 | 1 | -1 | 1 | 1 | 1 | -1 | 1 | 4 | Yes | Yes | Yes | No | Yes | Yes | Yes | Yes | Yes | Yes | Low |
| Smith, Tucker, Arnett, et al. 2013 | 1 | 1 | 1 | 1 | 1 | 1 | 0 | 1 | 7 | Yes | Yes | Yes | Yes | Yes | Yes | Yes | Yes | Yes | Yes | Low |
| Smith, Tucker, Lee, et al. 2013 | 1 | 1 | 1 | 1 | 1 | 1 | -1 | 1 | 6 | Yes | Yes | Yes | Yes | Yes | Yes | Yes | Yes | Yes | Yes | Low |
| Sonestedt et al. 2009 | 1 | 1 | 1 | 1 | 1 | 1 | 0 | 1 | 7 | Yes | Yes | Yes | Yes | Yes | Yes | Yes | Yes | Yes | Yes | Low |
| Sonestedt et al. 2011 | 1 | 1 | 1 | 1 | 1 | 1 | 1 | 1 | 8 | Yes | Yes | Yes | Yes | Yes | Yes | Yes | Yes | Yes | Yes | Low |
| Song et al. 2007 | 1 | 1 | 1 | 1 | 1 | 1 | -1 | 1 | 6 | Yes | Yes | Yes | Yes | No | Yes | Yes | Yes | Yes | Yes | Low |
| Sotos-Prieto et al. 2020 | 1 | 1 | 1 | 1 | 1 | 1 | 1 | 1 | 8 | Yes | Yes | Yes | Yes | Yes | Yes | Yes | Yes | Yes | Yes | Low |
| Tao et al. 2019 | 1 | 1 | 1 | 1 | 1 | 1 | 0 | 1 | 8 | Yes | Yes | Yes | Yes | Yes | Yes | Yes | Yes | Yes | Yes | Low |
| Vaughan et al. 2015 | 1 | 1 | 1 | 1 | -1 | 1 | -1 | 1 | 4 | Yes | Yes | Yes | Yes | Yes | Yes | Yes | Yes | Yes | Yes | Low |
| Vázquez-Moreno et al. 2020 | 1 | 1 | 1 | 1 | -1 | 1 | -1 | 1 | 4 | Yes | Yes | Yes | No | No | Yes | Yes | Yes | Yes | Yes | Low |
| Wang, Garcia-Bailo, et al. 2014 | 1 | 1 | 1 | 1 | -1 | 1 | 0 | 1 | 5 | Yes | Yes | Yes | Yes | Yes | Yes | Yes | Yes | Yes | Yes | Low |
| Wang, Tang, et al. 2014 | 1 | 1 | 1 | 1 | 1 | 1 | -1 | 1 | 6 | Yes | Yes | Yes | Yes | No | Yes | Yes | Yes | Yes | Yes | Low |
| Wang et al. 2015 | 1 | 1 | 1 | 1 | 1 | 1 | 0 | 1 | 7 | Yes | Yes | Yes | Yes | Yes | Yes | Yes | Yes | Yes | Yes | Low |
| Wang et al. 2017 | 1 | 1 | 1 | -1 | -1 | 1 | 1 | 1 | 4 | Yes | Yes | Yes | Yes | Yes | Yes | Yes | Yes | Yes | Yes | Low |
| Wang et al. 2018 | 1 | 1 | 1 | -1 | -1 | 1 | 1 | 1 | 4 | Yes | Yes | Yes | Yes | Yes | Yes | Yes | Yes | Yes | Yes | Low |
| Warodomwichit et al. 2009 | 1 | 1 | 1 | -1 | 1 | 1 | 0 | 1 | 5 | Yes | Yes | Yes | Yes | Yes | Yes | Yes | Yes | Yes | Yes | Low |
| Wu et al. 2017 | 1 | 1 | 1 | 1 | -1 | 1 | 1 | 1 | 6 | Yes | Yes | Yes | Yes | Yes | Yes | Yes | Yes | Yes | Yes | Low |
| Yarizadeh et al. 2021 | 1 | 1 | 1 | 1 | -1 | 1 | -1 | 1 | 4 | Yes | Yes | Yes | Yes | Yes | Yes | Yes | No | Yes | Yes | Low |
| Young et al. 2016 | 1 | 1 | 1 | 1 | 1 | 1 | 1 | 1 | 8 | Yes | Yes | Yes | Yes | Yes | Yes | Yes | Yes | Yes | Yes | Low |
| Zhang et al. 2015 | 1 | 1 | 1 | 1 | -1 | 1 | 0 | 1 | 5 | Yes | Yes | Yes | Yes | Yes | Yes | Yes | Yes | Yes | Yes | Low |
| Zhu, Xue, Guo, Deng, et al. 2020 | 1 | 1 | 1 | 1 | 1 | 1 | 0 | 1 | 7 | Yes | Yes | Yes | Yes | Yes | Yes | Yes | Yes | Yes | Yes | Low |
| Zhu, Xue, Guo and Yang 2020 | 1 | 1 | 1 | 1 | 1 | 1 | -1 | 1 | 6 | Yes | Yes | Yes | Yes | No | Yes | Yes | Yes | Yes | Yes | Low |
| **Interventional trials (n=60)** | | | | | | | | | | | | | | | | | | | | |
| Abete et al. 2009 | 1 | 1 | 1 | 1 | 1 | 1 | 0 | 1 | 7 | Yes | Yes | No | No | No | Yes | No | No | Yes | Yes | Neutral |
| Arias et al. 2017 | 1 | 1 | 1 | 1 | 1 | 1 | -1 | 1 | 6 | Yes | Yes | No | No | No | Yes | Yes | Yes | Yes | Yes | Neutral |
| Cha et al. 2006 | 1 | 1 | -1 | 1 | 1 | 1 | 0 | 1 | 5 | Yes | Yes | No | No | No | Yes | Yes | No | Yes | Yes | Neutral |
| Cha et al. 2007 | 1 | 1 | 1 | 1 | 1 | 1 | 1 | 1 | 8 | Yes | Yes | No | Yes | No | Yes | Yes | No | Yes | Yes | Neutral |
| Cha et al. 2014 | 1 | 1 | 1 | 1 | -1 | 1 | -1 | 1 | 4 | Yes | Yes | Yes | Yes | Yes | Yes | Yes | Yes | Yes | Yes | Low |
| De Luis, Aller, Izaola, Sagrado, et al. 2012 | 1 | 1 | 1 | 1 | 1 | -1 | 0 | 1 | 5 | Yes | Yes | No | Yes | No | Yes | Yes | Yes | Yes | Yes | Neutral |
| De Luis, Aller, Izaola, de la Fuente, et al. 2012 | 1 | 1 | 1 | 1 | 1 | -1 | 1 | 1 | 6 | Yes | Yes | No | Yes | No | Yes | Yes | Yes | Yes | Yes | Neutral |
| De Luis, Aller, Izaola, Conde, et al. 2013 | 1 | 1 | 1 | 1 | 1 | -1 | 0 | 1 | 5 | Yes | Yes | No | Yes | No | Yes | Yes | Yes | Yes | Yes | Neutral |
| De Luis, Aller, Izaola, Sagrado, et al. 2013 | 1 | 1 | 1 | 1 | 1 | -1 | 0 | 1 | 5 | Yes | Yes | No | Yes | No | Yes | Yes | Yes | Yes | Yes | Neutral |
| De Luis, Izaola, et al. 2013 | 1 | 1 | 1 | 1 | 1 | -1 | 0 | 1 | 5 | Yes | Yes | No | Yes | No | Yes | Yes | Yes | Yes | Yes | Neutral |
| De Luis et al. 2014 | 1 | 1 | 1 | 1 | 1 | -1 | 0 | 1 | 5 | Yes | Yes | No | Yes | No | Yes | Yes | Yes | Yes | Yes | Neutral |
| De Luis et al. 2015 | 1 | 1 | 1 | 1 | 1 | -1 | 0 | 1 | 5 | Yes | Yes | No | Yes | No | Yes | Yes | Yes | Yes | Yes | Neutral |
| De Luis et al. 2016a | 1 | 1 | 1 | 1 | 1 | -1 | 0 | 1 | 5 | Yes | Yes | No | Yes | No | Yes | Yes | Yes | Yes | Yes | Neutral |
| De Luis et al. 2016b | 1 | 1 | 1 | 1 | 1 | -1 | 0 | 1 | 5 | Yes | Yes | No | Yes | No | Yes | Yes | Yes | Yes | Yes | Neutral |
| De Luis, Izaola, et al. 2018 | 1 | 1 | 1 | 1 | 1 | -1 | 0 | 1 | 5 | Yes | Yes | No | Yes | No | Yes | Yes | Yes | Yes | Yes | Neutral |
| De Luis, Fernández Ovalle, et al. 2018 | 1 | 1 | 1 | 1 | 1 | -1 | 0 | 1 | 5 | Yes | Yes | No | Yes | No | Yes | Yes | Yes | Yes | Yes | Neutral |
| De Luis et al. 2019 | 1 | 1 | 1 | 1 | 1 | -1 | 0 | 1 | 5 | Yes | Yes | No | Yes | No | Yes | Yes | Yes | Yes | Yes | Neutral |
| Di Renzo et al. 2013 | 1 | 1 | 1 | 1 | -1 | -1 | -1 | 1 | 2 | Yes | Yes | No | Yes | No | Yes | Yes | No | Yes | Yes | Neutral |
| Di Renzo et al. 2018 | 1 | 1 | 1 | 0 | 1 | 1 | 0 | 1 | 6 | Yes | Yes | Yes | Yes | No | Yes | Yes | Yes | Yes | Yes | Low |
| Frankwich et al. 2016 | 1 | 1 | 0 | 0 | -1 | 1 | -1 | 1 | 2 | Yes | Yes | Yes | Yes | No | Yes | Yes | Yes | Yes | Yes | Low |
| Goni et al. 2018 | 1 | 1 | 1 | 1 | 1 | 1 | 0 | 1 | 7 | Yes | Yes | Yes | No | No | Yes | Yes | No | Yes | Yes | Low |
| Goni et al. 2019 | 1 | 1 | 1 | 1 | 1 | 1 | 1 | 1 | 8 | Yes | Yes | Yes | Yes | Yes | Yes | Yes | No | Yes | Yes | Low |
| Grau et al. 2009 | 1 | 1 | 1 | 0 | 1 | 1 | 0 | 1 | 6 | Yes | Yes | Yes | Yes | No | Yes | Yes | Yes | Yes | Yes | Low |
| Grau et al. 2010 | 1 | 1 | 1 | 1 | 1 | 1 | 1 | 1 | 8 | Yes | Yes | Yes | Yes | No | Yes | Yes | Yes | Yes | Yes | Low |
| Guinotte et al. 2003 | 1 | 1 | 1 | 1 | -1 | 1 | -1 | 1 | 4 | Yes | Yes | No | Yes | No | Yes | Yes | Yes | Yes | Yes | Neutral |
| Hamada et al. 2011 | 1 | 1 | 1 | 1 | 1 | -1 | -1 | 1 | 4 | Yes | Yes | No | No | No | Yes | Yes | No | Yes | Yes | Neutral |
| Heianza et al. 2016 | 1 | 1 | 1 | 1 | 1 | 1 | 1 | 1 | 8 | Yes | Yes | Yes | Yes | Yes | Yes | Yes | Yes | Yes | Yes | Low |
| Heianza et al. 2017 | 1 | 1 | 1 | 1 | 1 | 1 | 1 | 1 | 8 | Yes | Yes | Yes | Yes | Yes | Yes | Yes | Yes | Yes | Yes | Low |
| Hernandex-Guerrero et al. 2018 | 1 | 1 | -1 | 0 | -1 | 0 | 0 | 1 | 1 | Yes | Yes | No | Yes | No | Yes | Yes | Yes | Yes | Yes | Neutral |
| Huang et al. 2018 | 1 | 1 | 1 | -1 | 1 | 1 | 1 | 1 | 6 | Yes | Yes | Yes | Yes | Yes | Yes | Yes | No | Yes | Yes | Low |
| Labayen et al. 2015 | 1 | 0 | -1 | -1 | 1 | 1 | 0 | 1 | 2 | Yes | Yes | No | Yes | No | Yes | Yes | No | Yes | Yes | Neutral |
| Lee et al. 2012 | 1 | 1 | 1 | 1 | -1 | 1 | 0 | 1 | 5 | Yes | Yes | Yes | Yes | Yes | Yes | Yes | Yes | Yes | Yes | Low |
| Li et al. 2020 | 1 | 1 | 1 | 0 | 1 | 1 | 1 | 1 | 7 | Yes | Yes | Yes | Yes | Yes | Yes | Yes | Yes | Yes | Yes | Low |
| Lin et al. 2015 | 1 | 1 | 1 | 1 | 1 | 1 | 1 | 1 | 8 | Yes | Yes | Yes | No | Yes | Yes | Yes | Yes | Yes | Yes | Low |
| Lisboa et al. 2020 | 1 | 1 | 0 | 0 | -1 | 1 | -1 | 1 | 2 | Yes | Yes | Yes | Yes | Yes | Yes | Yes | Yes | Yes | Yes | Low |
| Mammes et al. 2001 | 1 | 1 | -1 | 1 | 1 | -1 | 0 | 1 | 3 | Yes | Yes | No | Yes | No | Yes | Yes | Yes | Yes | Yes | Neutral |
| Martinez-Lopez et al. 2013 | 1 | 1 | 1 | 1 | 1 | 1 | 0 | 1 | 7 | Yes | Yes | No | No | No | Yes | Yes | No | Yes | Yes | Neutral |
| Matsuo et al. 2009 | 1 | 1 | 1 | 1 | 1 | 1 | 0 | 1 | 7 | Yes | Yes | No | No | No | Yes | Yes | Yes | Yes | Yes | Neutral |
| Mattei et al. 2012 | 1 | 1 | 1 | 1 | 1 | 1 | 1 | 1 | 8 | Yes | Yes | Yes | Yes | Yes | Yes | Yes | Yes | Yes | Yes | Low |
| Namazi et al. 2017 | 1 | 1 | 1 | 0 | -1 | 1 | -1 | 1 | 3 | Yes | Yes | Yes | Yes | Yes | Yes | Yes | Yes | Yes | Yes | Low |
| Nikpay et al. 2020 | 1 | 1 | 1 | 1 | 1 | 1 | 1 | 1 | 8 | Yes | Yes | No | Yes | No | Yes | Yes | No | Yes | Yes | Neutral |
| Ramos-Lopez et al. 2019 | 1 | 1 | 1 | 1 | -1 | 1 | 0 | 1 | 5 | Yes | Yes | Yes | Yes | No | Yes | Yes | Yes | Yes | Yes | Low |
| Rauhio et al. 2013 | 1 | 1 | -1 | 1 | -1 | 1 | 0 | 1 | 3 | Yes | Yes | Unclear | Yes | Unclear | Yes | Yes | Yes | Yes | Yes | Low |
| Razquin et al. 2010 | 1 | 1 | 1 | 1 | 1 | 1 | 1 | 1 | 8 | Yes | Yes | Yes | Yes | Yes | Yes | Yes | Yes | Yes | Yes | Low |
| Rodrigues et al. 2018 | 1 | 1 | 1 | 1 | 1 | 1 | 0 | 1 | 7 | Yes | Yes | Yes | Yes | Yes | Yes | Yes | Yes | Yes | Yes | Low |
| Ruiz et al. 2011 | 1 | 1 | -1 | 1 | 1 | -1 | 0 | 1 | 3 | Yes | Yes | No | Yes | No | Yes | Yes | Yes | Yes | Yes | Neutral |
| San-Cristobal et al. 2017 | 1 | 1 | 1 | 1 | 1 | 1 | 1 | 1 | 8 | Yes | Yes | Yes | Yes | No | Yes | Yes | Yes | Yes | Yes | Low |
| Seip et al. 2008 | 1 | 1 | 1 | -1 | 1 | 1 | 0 | 1 | 5 | Yes | Yes | Yes | No | No | Yes | Yes | No | Yes | Yes | Low |
| Soenen et al. 2009 | 1 | 1 | -1 | 1 | 1 | 1 | 0 | 1 | 5 | Yes | Yes | No | Unclear | No | Yes | Yes | Yes | Yes | Yes | Neutral |
| Solis et al. 2008 | 1 | 1 | 1 | 1 | -1 | 1 | -1 | 1 | 4 | Yes | Yes | No | Yes | No | Yes | Yes | Yes | Yes | Yes | Neutral |
| Stocks et al. 2012 | 1 | 1 | 1 | 1 | 1 | 1 | 1 | 1 | 8 | Yes | Yes | Yes | Yes | No | Yes | Yes | Yes | Yes | Yes | Low |
| Svendstrup et al. 2018 | 1 | 1 | 1 | 1 | 1 | 1 | 1 | 1 | 8 | Yes | Yes | Yes | Yes | No | Yes | Yes | Yes | Yes | Yes | Low |
| Tan et al. 2020 | 1 | 1 | 1 | 0 | 1 | 1 | 0 | 1 | 6 | Yes | Yes | Yes | Yes | No | Yes | Yes | Yes | Yes | Yes | Low |
| Teixeira et al. 2020 | 1 | 1 | 1 | 1 | 1 | 1 | 0 | 1 | 7 | Yes | Yes | No | Yes | No | Yes | Yes | No | Yes | Yes | Neutral |
| Teixeira et al. 2020 | 1 | 1 | 1 | 1 | 1 | 1 | 0 | 1 | 7 | Yes | Yes | No | Yes | No | Yes | Yes | No | Yes | Yes | Neutral |
| Tsuzaki et al. 2009 | 1 | 1 | -1 | 1 | 1 | -1 | -1 | 1 | 2 | Yes | Yes | No | No | No | Yes | Yes | No | Yes | Yes | Neutral |
| Verhoef et al. 2014 | 1 | 1 | 1 | 1 | 1 | 0 | 0 | 1 | 6 | Yes | Yes | No | Yes | No | Yes | Yes | Yes | Yes | Yes | Neutral |
| Xinli et al. 2001 | 1 | 1 | 1 | 1 | 1 | 1 | 0 | 1 | 7 | Yes | Yes | No | Yes | No | Yes | Yes | Yes | Yes | Yes | Neutral |
| Yoon et al. 2007 | 1 | 1 | -1 | 1 | -1 | 1 | 1 | 1 | 4 | Yes | Yes | No | No | No | Yes | Yes | No | Yes | Yes | Neutral |
| Zhang et al. 2012 | 1 | 1 | 1 | 1 | 1 | 1 | 1 | 1 | 8 | Yes | Yes | Yes | No | Yes | Yes | Yes | Yes | Yes | Yes | Low |
