# Supplementary material for: In the context of the triple burden of malnutrition: A systematic review of gene-diet interactions and nutritional status
Source: Crit Rev Food Sci Nutr. 2022 Oct 12;64(11):3235–63. doi: 10.1080/10408398.2022.2131727 (PMC11000749; doi:10.1080/10408398.2022.2131727)
Supplement: Supplemental Material [file BFSN_A_2131727_SM9779.zip › Supp/Tan_Supplementary_270922.pdf]

SUPPLEMENTARY TABLES

In the Context of the Triple Burden of Malnutrition: A Systematic Review of Gene-Diet Interactions and Nutritional Status

Pui Yee Tan, J Bernadette Moore, Ling Bai, GuYuan Tang, YunYun Gong*

*Corresponding author: YunYun Gong, y.gong@leeds.ac.uk

Table of Contents

Table S1: Search strategies developed for each database 2

Table S2: Scoring system for assessing methodological quality of gene-diet interaction studies 11

Table S3: Risk of bias assessment outcomes for all studies reviewed (n=168) 12

Table S1: Search strategies developed for each database

PubMed

Searched 30th April 2021

No.	Concept	Keywords	Results
#52	Filter [non-human studies]	Search: #47 NOT #51	1,204
#51		Search: #48 OR #49 OR #50	3,329,227
#50		Search: ((((((((((((((broiler?[Title/Abstract]) OR (piglet?[Title/Abstract])) OR (horse[Title/Abstract])) OR (cattle[Title/Abstract])) OR (primate?[Title/Abstract])) OR (mice[Title/Abstract])) OR (rat[Title/Abstract])) OR (rats[Title/Abstract])) OR (mouse[Title/Abstract])) OR (murine[Title/Abstract])) OR (pigs[Title/Abstract])) OR (pig[Title/Abstract])) OR (cow[Title/Abstract])) OR (sheep[Title/Abstract])) OR (hens[Title/Abstract])) OR (hen[Title/Abstract])	3,147,004
#49		Search: "Animal Experimentation"[Mesh] Sort by: Most Recent	9,634
#48		Search: "Models, Animal"[Mesh] Sort by: Most Recent	586,642
#47	Gene-diet interaction on nutritional status	Search: #20 AND #46	20,317
#46	Nutritional status	Search: #26 OR #45	1,418,743
#45		Search: #32 OR #36 OR #40 OR #44	41,986
#44		Search: #42 OR #43	20,999
#43		Search: "Anemia, Iron-Deficiency"[Mesh] Sort by: Most Recent	10,269
#42		Search: #27 AND #28 AND #41	14,397
#41		Search: (((iron[Title/Abstract]) OR (ferritin[Title/Abstract])) OR (transferrin[Title/Abstract])) OR (h?emoglobin[Title/Abstract])	224,438
#40		Search: #38 OR #39	9,652
#39		Search: "Folic Acid Deficiency"[Mesh] Sort by: Most Recent	4,962
#38		Search: #27 AND #28 AND #37	5,902
#37		Search: ((folate[Title/Abstract]) OR ("folic acid"[Title/Abstract])) OR ("vitamin B12"[Title/Abstract])	54,691
#36		Search: #34 OR #35	7,463
#35		Search: "Vitamin A Deficiency"[Mesh] Sort by: Most Recent	5,782
#34		Search: #27 AND #28 AND #33	2,993
#33		Search: ("vitamin A"[Title/Abstract]) OR (retinol[Title/Abstract])	34,646
#32		Search: #30 OR #31	8,102
#31		Search: "Zinc/deficiency"[Mesh] Sort by: Most Recent	4,961
#30		Search: #27 AND #28 AND #29	4,573
#29		Search: zinc[Title/Abstract]	123,230

#28		Search: (((((deficient[Title/Abstract]) OR (deficienc*[Title/Abstract])) OR (inadequate[Title/Abstract])) OR (inadequac*[Title/Abstract])) OR (insufficient[Title/Abstract])) OR (insufficienc*[Title/Abstract])	851,853
#27		Search: ((serum[Title/Abstract]) OR (plasma[Title/Abstract])) OR (blood[Title/Abstract])	3,425,317
#26		Search: #21 OR #22 OR #23 OR #24 OR #25	1,394,644
#25		Search: (((((weight[Title/Abstract]) OR ("body mass index"[Title/Abstract])) OR (BMI[Title/Abstract])) OR ("height-for-age"[Title/Abstract])) OR ("weight-for-height"[Title/Abstract])) OR ("weight-for-age"[Title/Abstract])	1,037,397
#24		Search: ((((((((((obese[Title/Abstract]) OR (obesity[Title/Abstract])) OR (overweight[Title/Abstract])) OR (over?nutrition[Title/Abstract])) OR (under?nutrition[Title/Abstract])) OR (stunting[Title/Abstract])) OR (stunted[Title/Abstract])) OR (underweight[Title/Abstract])) OR (malnutrition[Title/Abstract])) OR (wasting[Title/Abstract])) OR (adiposity[Title/Abstract])	414,448
#23		Search: "Malnutrition"[Mesh] OR "Protein-Energy Malnutrition"[Mesh] Sort by: Most Recent	124,067
#22		Search: "Overweight"[Mesh] Sort by: Most Recent	227,654
#21		Search: "Obesity"[Mesh] Sort by: Most Recent	220,988
#20	Gene-diet interaction	Search: #13 AND #19	79,797
#19		Search: #14 OR #15 OR #16 OR #17 OR #18	1,445,031
#18		Search: "Diet"[Mesh] Sort by: Most Recent	289,703
#17		Search: "Food"[Mesh] Sort by: Most Recent	635,227
#16		Search: "Nutrients"[Mesh] Sort by: Most Recent	56,543
#15		Search: (((((diet?[Title/Abstract]) OR (dietary[Title/Abstract])) OR (intake?[Title/Abstract])) OR (consumption?[Title/Abstract])) OR (meal?[Title/Abstract])) OR ("eating pattern?"[Title/Abstract])	918,757
#14		Search: "Eating"[Mesh] Sort by: Most Recent	73,835
#13		Search: #7 OR #12	1,688,869
#12		Search: #9 OR #10 OR #11	981,416
#11		Search: "Gene Expression"[Mesh] Sort by: Most Recent	460,628
#10		Search: (("gene expression?"[Title/Abstract]) OR (microRNA?[Title/Abstract])) OR (miRNA?[Title/Abstract])	498,399
#9		Search: (((epigenetic?[Title/Abstract]) OR (methylation?[Title/Abstract])) OR (acetylation?[Title/Abstract])) OR ("histone modification?"[Title/Abstract])	174,984
#8		Search: "Epigenomics"[Mesh] Sort by: Most Recent	6,001
#7		Search: #1 OR #2 OR #3 OR #6	815,237
#6		Search: #4 AND #5	580,318

#5		Search: ((((variant?[Title/Abstract]) OR (variation?[Title/Abstract])) OR (mutant?[Title/Abstract])) OR (mutation?[Title/Abstract])) OR (polymorphism?[Title/Abstract])	1,397,376
#4		Search: (gene?[Title/Abstract]) OR (genetic?[Title/Abstract])	2,347,190
#3		Search: ((allele?[Title/Abstract]) OR (genotype?[Title/Abstract])) OR ("polygenic risk"[Title/Abstract])	306,121
#2		Search: (("single nucleotide polymorphism?"[Title/Abstract]) OR (SNPs[Title/Abstract])) OR (SNP[Title/Abstract])	107,048
#1		Search: "Polymorphism, Single Nucleotide"[Mesh] Sort by: Most Recent	120,873

Embase

Searched 30th April 2021

No.	Theme	Search	Results
1	Gene-diet interaction	("single nucleotide polymorphism?" or SNP or SNPs).ti,ab,kw.	169,983
2		single nucleotide polymorphism/	197,532
3		(allele? or genotype? or "polygenic risk").ti,ab,kw.	533,093
4		(gene or genes or genetic or genetics).ti,ab,kw.	3,068,934
5		(variant? or variation? or mutant? or mutation? or polymorphism?).ti,ab,kw.	2,235,621
6		4 and 5	1,077,999
7		1 or 2 or 3 or 6	1,377,305
8		(epigenetic? or methylation? or "acetylation?" or "histone modification?").ti,ab,kw.	224,213
9		("gene expression?" or microRNA? or miRNA?).ti,ab,kw.	660,238
10		epigenetics/	82,951
11		8 or 9 or 10	844,524
12		7 or 11	2,067,918
13		dietary pattern/ or dietary intake/	80,764
14		nutrient intake/	994
15		(diet? or dietary or intake? or consumption? or meal? or "eating pattern?").ti,ab,kw.	1,027,017
16		13 or 14 or 15	1,042,449
17		12 and 16	78,885
18	Nutritional status	obesity/dm, ep, et, pc [Disease Management, Epidemiology, Etiology, Prevention]	44,083
19		malnutrition/dm, ep, et, pc [Disease Management, Epidemiology, Etiology, Prevention]	6,258
20		protein calorie malnutrition/dm, ep, et, pc [Disease Management, Epidemiology, Etiology, Prevention]	791
21		stunting/dm, ep, et, pc [Disease Management, Epidemiology, Etiology, Prevention]	325
22		underweight/dm, ep, et, pc [Disease Management, Epidemiology, Etiology, Prevention]	656

23		(obese or overweight or obesity or over?nutrition or under?nutrition or stunting or stunted or underweight or wasting or malnutrition).ti,ba,kw.	248,530
24		(weight or "body mass index" or BMI or "height-for-age" or "weight-for-age" or "weight-for-height" or adiposity).ti,ab,kw.	1,289,441
25		18 or 19 or 20 or 21 or 22 or 23 or 24	1,400,127
26		(serum or plasma or blood).ti,ab,kw.	3,449,020
27		("deficient" or "deficienc*" or "insufficient" or "insufficienc*" or "inadequate" or "inadequac*").ti,ab,kw.	930,883
28		zinc.ti,ab,kw.	116,617
29		26 and 27 and 28	5,104
30		zinc deficiency/dm, ep, et, pc [Disease Management, Epidemiology, Etiology, Prevention]	485
31		29 or 30	5,417
32		("vitamin A" or retinol).ti,ab,kw.	26,662
33		26 and 27 and 32	2,933
34		retinol deficiency/dm, ep, et, pc [Disease Management, Epidemiology, Etiology, Prevention]	1,042
35		33 or 34	3,626
36		(folate or "folic acid" or "vitamin B12").ti,ab,kw.	55,961
37		26 and 27 and 36	7,863
38		folic acid deficiency/dm, ep, et, pc [Disease Management, Epidemiology, Etiology, Prevention]	789
39		37 or 38	8,389
40		(iron or ferritin or h?emoglobin or transferrin).ti,ab,kw.	392,899
41		26 and 27 and 40	26,374
42		iron deficiency/dm, ep, et, pc [Disease Management, Epidemiology, Etiology, Prevention]	1,272
43		iron deficiency anemia/dm, ep, et, pc [Disease Management, Epidemiology, Etiology, Prevention]	3,285
44		41 or 42 or 43	29,233
45		31 or 35 or 39 or 44	40,731
46		25 or 45	1,434,053
47	Gene-diet interaction on nutritional status	17 and 46	20,864
48	Filter [non-human studies, non-healthy population with diseases]	animal experiment/	1,856,677
49		47 not 48	13,337
50		animal model/	1,300,883
51		49 not 50	12,934
52		(broiler? or cattle or piglet? or primate? or horse).ti,ab,kw.	192,680
53		51 not 52	12,481
54		(mice or mouse or rat or rats or "murine model" or "animal model" or "pig model" or "rodent model").ti,ab,kw.	2,360,409

55		53 not 54	9,151
56		limit 55 to article	4,976
57		(pig or pigs or hens or cow).ti,ab,kw.	171,469
58		56 not 57	4,783
59		limit 58 to elderly - focused	58
60		58 not 59	4,725
61		limit 60 to animals	505
62		60 not 61	4,220
63		malignant neoplasm/	60,350
64		62 not 63	4,213
65		("breast cancer" or "liver cancer" or "lung cancer" or "colon cancer" or "colorectal cancer" or Alzhemier's).ti,ab,kw.	824,586
66		64 not 65	4,024
67		("liver disease" or fibrosis).ti,ab,kw.	382,061
68		66 not 67	3,846

Scopus

Searched 30th April 2021

No.	Theme	Search	Results
35	Filter [non-human studies and elderly]	#31 AND NOT #34	1,967
34		TITLE-ABS-KEY ((elderly OR "older-adult?" OR "elder-adult?"))	807,657
33		#31 AND NOT #32	2,027
32		TITLE-ABS-KEY ((primate? OR broiler? OR cattle OR cow OR cows OR sheep OR sheeps OR hens OR mice OR mouse OR rat OR rats OR "animal-model?" OR murine OR pig OR pigs OR piglet? OR horse OR rodent OR swine))	5,241,476
31		#31 Filter Article only	3,330
30	Gene-diet interaction on nutritional status	#27 AND #29	4,246
29		#12 OR #28	13,196
28		TITLE-ABS-KEY (“gene-diet-interaction?”)	275
27	Nutritional status	#15 OR #26	3,140,441
26		#19 OR #21 OR #23 OR #25	56,985
25		#16 AND #17 AND #24	36,810
24		TITLE-ABS-KEY ((iron OR ferritin OR transferrin OR h?emoglobin))	999,756
23		#16 AND #17 AND #22	13,417
22		TITLE-ABS-KEY ((folate OR "folic-acid" OR "vitamin-B12"))	99,072
21		#16 AND #17 AND #20	6,403

20		TITLE-ABS-KEY (("vitamin-A" OR retinol))	70,935
19		#16 AND #17 AND #18	10,167
18		TITLE-ABS-KEY (zinc)	589,890
17		TITLE-ABS-KEY ((deficient OR deficienc* OR inadequate OR inadequac* OR insufficient OR insufficienc*))	1,766,54 5
16		TITLE-ABS-KEY ((serum OR plasma OR blood))	6,633,26 5
15		#13 OR #14	3,094,84 5
14		TITLE-ABS-KEY ((weight OR adiposity OR “body-mass-index” OR bmi OR "height-for-age" OR "weight-for-age" OR "weight-for-height"))	2,749,80 2
13		TITLE-ABS-KEY ((obese OR obesity OR overweight OR over?nutrition OR under?nutrition OR underweight OR stunting OR stunted OR wasting OR malnutrition))	643,195
12	Gene-diet interaction	#10 AND #11	13,067
11		TITLE-ABS-KEY ((diet? OR dietary OR intake? OR meal? OR consumption? OR "eating-pattern?"))	676,938
10		#6 OR #9	1,080,91 5
9		#7 OR #8	202,790
8		TITLE-ABS-KEY ((“gene-expression?” OR mirna? OR microrna?))	124,587
7		TITLE-ABS-KEY ((epigenetic? OR methylation? OR acetylation? OR "histone-modification?"))	83,662
6		#1 OR #2 OR #5	895,333
5		#3 AND #4	550,698
4		TITLE-ABS KEY ((variant? OR variation? OR mutant? OR mutation? OR polymorphism?))	2,038,52 6
3		TITLE-ABS-KEY ((gene? OR genetic?))	3,009,35 2
2		TITLE-ABS-KEY ((allele? OR genotype? OR "polygenic-risk"))	398,926
1		TITLE-ABS-KEY ((“single-nucleotide-polymorphism?” OR snp OR snps))	130,446

Web of Science

Searched 30th April 2021

No.	Theme	Search	Results
# 36		#33 NOT #34	4,864

	Filter [non-human studies, non-related field, review articles, elderly]	Refined by: [excluding] WEB OF SCIENCE CATEGORIES: (WATER RESOURCES OR PLANT SCIENCES OR ENERGY FUELS OR BIOPHYSICS OR DERMATOLOGY OR EVOLUTIONARY BIOLOGY OR UROLOGY NEPHROLOGY OR SOIL SCIENCE OR MARINE FRESHWATER BIOLOGY OR VIROLOGY OR CHEMISTRY APPLIED OR ALLERGY OR ENGINEERING CHEMICAL OR SUBSTANCE ABUSE OR CELL TISSUE ENGINEERING OR AGRONOMY OR DENTISTRY ORAL SURGERY MEDICINE OR GERONTOLOGY OR SOCIAL SCIENCES BIOMEDICAL OR TRANSPLANTATION OR SURGERY OR CRITICAL CARE MEDICINE OR ECONOMICS OR ELECTROCHEMISTRY OR ENGINEERING BIOMEDICAL OR ORTHOPEDICS OR RHEUMATOLOGY OR TROPICAL MEDICINE OR GREEN SUSTAINABLE SCIENCE TECHNOLOGY OR HORTICULTURE OR PARASITOLOGY OR PSYCHOLOGY DEVELOPMENTAL OR CHEMISTRY ANALYTICAL OR WOMEN S STUDIES OR INFECTIOUS DISEASES OR ANATOMY MORPHOLOGY OR ANESTHESIOLOGY OR CHEMISTRY ORGANIC OR CHEMISTRY PHYSICAL OR COMPUTER SCIENCE INTERDISCIPLINARY APPLICATIONS OR GEOSCIENCES MULTIDISCIPLINARY OR ZOOLOGY OR HISTORY PHILOSOPHY OF SCIENCE) AND [excluding] WEB OF SCIENCE CATEGORIES: (ONCOLOGY OR AGRICULTURE DAIRY ANIMAL SCIENCE OR GASTROENTEROLOGY HEPATOLOGY OR PHARMACOLOGY PHARMACY OR VETERINARY SCIENCES) AND [excluding] WEB OF SCIENCE CATEGORIES: (PSYCHIATRY OR TOXICOLOGY) AND [excluding] WEB OF SCIENCE CATEGORIES: (ANDROLOGY OR HOSPITALITY LEISURE SPORT TOURISM OR GERIATRICS GERONTOLOGY OR OTORHINOLARYNGOLOGY OR POLYMER SCIENCE OR PRIMARY HEALTH CARE OR ARCHAEOLOGY OR BIODIVERSITY CONSERVATION OR CONSTRUCTION BUILDING TECHNOLOGY OR EDUCATION SCIENTIFIC DISCIPLINES OR ENGINEERING CIVIL OR ENGINEERING MULTIDISCIPLINARY OR ETHNIC STUDIES OR RESPIRATORY SYSTEM OR HEALTH POLICY SERVICES OR ENTOMOLOGY OR INFORMATION SCIENCE LIBRARY SCIENCE OR NURSING OR MANAGEMENT OR MATERIALS SCIENCE MULTIDISCIPLINARY OR MATERIALS SCIENCE PAPER WOOD OR MATERIALS SCIENCE TEXTILES OR MECHANICS OR MEDICAL INFORMATICS OR PHYSICS APPLIED OR NEUROIMAGING OR REHABILITATION OR PSYCHOLOGY EXPERIMENTAL OR SPECTROSCOPY OR RADIOLOGY NUCLEAR MEDICINE MEDICAL IMAGING OR URBAN STUDIES) AND [excluding] WEB OF SCIENCE CATEGORIES: (OPHTHALMOLOGY OR AGRICULTURE MULTIDISCIPLINARY OR CLINICAL NEUROLOGY OR ECOLOGY OR NEUROSCIENCES OR PSYCHOLOGY)	

		AND [excluding] WEB OF SCIENCE CATEGORIES: (MICROBIOLOGY)	
# 35		#33 NOT #34	6,787
# 34		TS=(animal-model$ OR animal-studies OR animal-study OR mice OR mouse OR mice-model OR rat OR rats OR rat-model OR murine-model OR pig-model OR rodent-model OR primate$ OR broiler$ OR horse$ OR cattle OR swine-model)	3,829,477
# 33		#31 NOT #32	17,913
# 32		TS=(older-adult$ OR elderly OR elders)	453,223
# 31		#29 AND #14 Refined by: DOCUMENT TYPES: (ARTICLE)	18,164
# 30	Gene-diet interaction on nutritional status	#29 AND #14	21,194
# 29	Nutritional status	#28 OR #17	1,255,652
# 28		#27 OR #25 OR #23 OR #21	34,480
# 27		#26 AND #19 AND #18	6,946
# 26		TS=(folate OR vitamin-B12 OR folic-acid)	65,569
# 25		#24 AND #19 AND #18	3,633
# 24		TS=(vitamin-A OR retinol)	47,406
# 23		#22 AND #19 AND #18	22,665
# 22		TS=(iron OR ferritin OR transferrin OR h$emoglobin)	816,097
# 21		#20 AND #19 AND #18	6,981
# 20		TS=zinc	359,648
# 19		TS=(deficien* OR inadequa* OR insufficien*)	1,136,652
# 18		TS=(serum OR plasma OR blood)	4,068,993
# 17		#16 OR #15	1,225,352
# 16		TS=(obese OR obesity OR overweight OR over$nutrition OR under$nutrition OR stunting OR stunted OR underweight OR wasting OR malnutrition)	996,421
# 15		TS=(weigh OR body-mass-index OR BMI OR height-for-age OR weight-for-age OR weight-for-height OR adiposity)	383,727
# 14	Gene-diet interaction	#13 AND #11	90,231
# 13		TS=(diet$ OR dietary OR intake$ OR consumption$ OR meal$ OR dietary-pattern$ OR eating-pattern$)	1,672,291
# 12		TS=(diet$ OR dietary OR intake$ OR consumption$ OR meal$ OR dietary-pattern$)	1,671,424
# 11		#10 OR #7	2,318,722
# 10		#9 OR #8	947,387

# 9		TS=(epigentic$ OR DNA-methylation$ OR acetylation$ OR histone-modification$)	120,247
# 8		TS=(miRNA$ OR microRNA$ OR gene-expression$)	863,319
# 7		#6 OR #3 OR #2 OR #1	1,538,424
# 6		#5 AND #4	1,226,082
# 5		TS=(variant$ OR variation$ OR mutant$ OR mutation$ OR polymorphism$)	3,615,956
# 4		TS=(gene$ OR genetic$)	3,884,198
# 3		TS=polygenic-risk	2,401
# 2		TS=(allele$ OR genotype$)	580,029
# 1		TS=(single-nucleotide-polymorphism$ OR SNP$)	148,472

Table S2: Scoring system for assessing methodological quality of gene-diet interaction studies

**Criteria**	**Low quality**	**Intermediate quality**	**High quality**
**Interaction as primary study goal**	No = -1	Not known = 0	Yes = 1
**Formal test for interaction**	No = -1	Not known or stratified analysis= 0	Yes = 1
**Correction for multiple testing**	No = -1	Not known = 0	Yes or not necessary = 1
**Correction for population stratification/ethnicity**	No = -1	Not known = 0	Yes or not applicable=1
**Hardy-Weinberg equilibrium**	No or not stated = -1	-	Yes = 1
**Group similarity at baseline tested**	No = -1	Not known = 0	Yes = 1
**Power analysis and sample size**			
**Observational studies**	<1000 = -1	1000-5000 = 0	>5000 = 1; or power analysis is provided (>80%)
***Intervention trials**	≤75=-1	76-365=0	>365= 1; or power analysis is provided (>80%)
**Sufficient details of study procedure stated**	No = -1	-	Yes = 1

*the cut offs used to define low, intermediate and high sufficiency of sample size were based on the 15th (n=75) and 75th percentile (n=365) of the sample size of the studies included.

Table S3: Risk of bias assessment outcomes for all studies reviewed (n=168)

**Author (years)**	**Methodological quality assessment for gene-diet interaction research**									**Quality Criteria Checklist**										
	**Interaction as primary study goal**	**Statistical test for interaction**	**Correction for multiple testing**	**Correction for ethnicity**	**Hardy-Weinberg Equilibrium**	**Group similarity at baseline tested**	**Sample size/power analysis**	**Sufficient study details**	**Score (full =8)**	**Research Question**	**No selection bias**	**Randomisation / group comparability**	**Withdrawal description**	**Blinding**	**Intervention/ exposure clearly described**	**Validity and reliability of outcome measures**	**Statistical analysis**	**Conclusion supported by results**	**No funding bias**	**Overall**
**Observational studies (n=108)**																				
Alathari et al 2021	1	1	1	1	1	1	-1	1	6	Yes	Yes	Yes	Yes	Yes	Yes	Yes	Yes	Yes	Yes	Low
Alsulami, Nyakotey, et al 2020	1	1	1	1	1	1	-1	1	6	Yes	Yes	Yes	Yes	Yes	Yes	Yes	Yes	Yes	Yes	Low
Alsulami, Aji, et al 2020	1	1	1	1	1	1	-1	1	6	Yes	Yes	Yes	Yes	Yes	Yes	Yes	Yes	Yes	Yes	Low
Ankarfeldt et al 2014	1	1	1	1	-1	1	1	1	6	Yes	Yes	Yes	Yes	Yes	Yes	Yes	Yes	Yes	Yes	Low
Barchitta et al 2014	1	1	1	1	1	1	-1	1	6	Yes	Yes	Yes	Yes	Yes	Yes	Yes	No	Yes	Yes	Low
Bauman-fortin et al 2019	1	1	1	1	1	1	-1	1	6	Yes	Yes	Yes	Yes	Yes	Yes	Yes	Yes	Yes	Yes	Low
Cade et al 2015	1	1	1	0	-1	1	0	1	4	Yes	Yes	Yes	Yes	Yes	Yes	Yes	Yes	Yes	Yes	Low
Casas-Agustench et al 2014	1	1	1	1	1	1	0	1	7	Yes	Yes	Yes	Yes	Yes	Yes	Yes	Yes	Yes	Yes	Low
Celis-Morales et al 2017	1	1	1	1	1	1	1	1	8	Yes	Yes	Yes	Yes	Yes	Yes	Yes	Yes	Yes	Yes	Low
Chen et al 2019	1	1	1	1	1	1	1	1	8	Yes	Yes	Yes	Yes	Yes	Yes	Yes	Yes	Yes	Yes	Low
Corella et al 2007	1	1	-1	1	1	1	0	1	5	Yes	Yes	Yes	Yes	Yes	Yes	Yes	Yes	Yes	Yes	Low
Corella et al 2009	1	1	1	1	1	1	0	1	7	Yes	Yes	Yes	Yes	Yes	Yes	Yes	Yes	Yes	Yes	Low

Corella et al 2011	1	1	1	1	-1	1	1	1	7	Yes	Yes	Yes	Yes	Yes	Yes	Yes	Yes	Yes	Yes	Low
Cummings et al 2017	1	1	1	0	-1	1	-1	1	3	Yes	Yes	Yes	No	No	Yes	Yes	Yes	Yes	Yes	Low
Czajkowski et al 2020	1	1	-1	1	1	1	-1	1	4	Yes	Yes	Yes	Yes	Yes	Yes	Yes	Yes	Yes	Yes	Low
Davis et al 2010	1	1	1	1	1	1	-1	1	6	Yes	Yes	Yes	Yes	No	Yes	Yes	Yes	Yes	Yes	Low
Dedoussis et al 2010	1	1	1	1	1	1	-1	1	6	Yes	Yes	Yes	Yes	Yes	Yes	Yes	Yes	Yes	Yes	Low
Dedoussis et al 2011	1	1	1	1	1	1	1	1	8	Yes	Yes	Yes	Yes	Yes	Yes	Yes	Yes	Yes	Yes	Low
Domínguez-Reyes et al 2015	1	1	-1	1	1	1	-1	1	4	Yes	Yes	Yes	Yes	Yes	Yes	Yes	Yes	Yes	Yes	Low
Doo et al 2014	1	1	1	1	1	1	1	1	8	Yes	Yes	Yes	Yes	Yes	Yes	Yes	Yes	Yes	Yes	Low
Doo & Kim 2010	1	1	1	1	1	1	0	1	7	Yes	Yes	Yes	Yes	Yes	Yes	Yes	Yes	Yes	Yes	Low
Dumont et al 2018	1	1	1	1	1	1	0	1	7	Yes	Yes	Yes	Yes	Yes	Yes	Yes	Yes	Yes	Yes	Low
Galmes et al 2020	1	1	1	0	-1	1	-1	1	3	Yes	Yes	Yes	Yes	No	Yes	Yes	Yes	Yes	Yes	Low
Garaulet et al 2014	1	1	1	1	-1	1	0	1	5	Yes	Yes	Yes	Yes	Yes	Yes	Yes	Yes	Yes	Yes	Low
Garske et al 2019	1	1	1	1	-1	1	1	1	6	Yes	Yes	Yes	Yes	Yes	Yes	Yes	Yes	Yes	Yes	Low
Gong et al 2021	1	1	-1	1	1	1	0	1	5	Yes	Yes	Yes	Yes	Yes	Yes	Yes	Yes	Yes	Yes	Low
Goni et al 2015	1	1	1	1	1	1	-1	1	6	Yes	Yes	Yes	Yes	No	Yes	Yes	Yes	Yes	Yes	Low
Goodarzi et al 2021	1	1	-1	1	1	1	0	1	5	Yes	Yes	Yes	Yes	Yes	Yes	Yes	Yes	Yes	Yes	Low
Goodwin et al 2015	1	1	1	1	1	1	-1	1	6	Yes	Yes	Yes	Yes	No	Yes	Yes	Yes	Yes	Yes	Low
Han et al 2020	1	1	1	1	-1	1	1	1	6	Yes	Yes	Yes	Yes	Yes	Yes	Yes	Yes	Yes	Yes	Low
Higashibata et al 2016	1	1	1	1	1	1	0	1	7	Yes	Yes	Yes	Yes	Yes	Yes	Yes	Yes	Yes	Yes	Low
Hiraoka 2004	1	1	1	1	-1	1	-1	1	4	Yes	Yes	Yes	Yes	No	Yes	Yes	Yes	Yes	Yes	Low

Hiroi et al 2011	1	1	1	1	-1	1	0	1	5	Yes	Yes	Yes	Yes	Yes	Yes	Yes	Yes	Yes	Yes	Low
Hosseini-Esfabani et al 2019	1	1	1	1	1	1	0	1	7	Yes	Yes	Yes	Yes	Yes	Yes	Yes	Yes	Yes	Yes	Low
Hosseini-Esfahani et al 2017	1	1	1	1	1	1	0	1	7	Yes	Yes	Yes	Yes	Yes	Yes	Yes	Yes	Yes	Yes	Low
Huang et al 2015	1	1	1	1	1	1	1	1	8	Yes	Yes	Yes	Yes	Yes	Yes	Yes	Yes	Yes	Yes	Low
Huang, Wang, Heianza, Zheng, et al 2019	1	1	1	1	-1	1	1	1	6	Yes	Yes	Yes	Yes	Yes	Yes	Yes	Yes	Yes	Yes	Low
Huang, Wang, Heianza, Wiggs, et al 2019	1	1	1	1	1	1	1	1	8	Yes	Yes	Yes	Yes	Yes	Yes	Yes	Yes	Yes	Yes	Low
Huriyati et al 2016	1	-1	1	1	-1	1	-1	1	2	Yes	Yes	Yes	Yes	No	Yes	No	No	Yes	Yes	Neutral
Huriyati et al 2020	1	1	1	1	-1	1	-1	1	4	Yes	Yes	Yes	Yes	No	Yes	Yes	Yes	Yes	Yes	Low
Jaaskelainen et al 2013	1	0	1	1	1	1	0	1	6	Yes	Yes	Yes	Yes	Yes	Yes	Yes	No	Yes	Yes	Low
Jaaskelainen et al 2013	1	0	1	1	1	1	0	1	6	Yes	Yes	Yes	Yes	Yes	Yes	Yes	No	Yes	Yes	Low
Jiang et al 2019	1	1	1	1	1	1	0	1	7	Yes	Yes	Yes	Yes	Yes	Yes	No	Yes	Yes	Yes	Neutral
Joffe et al 2014	1	1	1	1	1	1	-1	1	6	Yes	Yes	Yes	Yes	Yes	Yes	Yes	Yes	Yes	Yes	Low
Junyent et al 2010	1	1	-1	1	1	1	-1	1	4	Yes	Yes	Yes	Yes	Yes	Yes	Yes	Yes	Yes	Yes	Low
Kokaze et al 2014	1	1	1	1	-1	1	-1	1	4	Yes	Yes	Yes	Yes	No	Yes	Yes	Yes	Yes	Yes	Low
Labayen et al 2016	1	1	1	-1	1	1	-1	1	4	Yes	Yes	Yes	Yes	Yes	Yes	Yes	Yes	Yes	Yes	Low
Larsen et al 2014	1	1	1	1	-1	1	1	1	6	Yes	Yes	Yes	Yes	Yes	Yes	Yes	Yes	Yes	Yes	Low
Larsen et al 2014	1	1	1	1	-1	1	1	1	6	Yes	Yes	Yes	Yes	Yes	Yes	Yes	Yes	Yes	Yes	Low
Latella et al 2009	1	1	1	1	1	1	-1	1	6	Yes	Yes	Yes	Yes	Yes	Yes	Yes	Yes	Yes	Yes	Low

Lee et al 2017	1	1	1	1	1	1	-1	1	6	Yes	Yes	Yes	Yes	No	Yes	Yes	Yes	Yes	Yes	Low
Lee et al 2020	1	1	1	1	1	1	1	1	8	Yes	Yes	Yes	Yes	Yes	Yes	Yes	Yes	Yes	Yes	Low
Lemas et al 2012	1	1	1	1	1	1	0	1	7	Yes	Yes	Yes	Yes	Yes	Yes	Yes	Yes	Yes	Yes	Low
Li et al 2019	1	1	1	0	1	1	1	1	7	Yes	Yes	Yes	Yes	Yes	Yes	Yes	Yes	Yes	Yes	Low
Lim et al 2014	1	1	1	1	1	1	0	1	7	Yes	Yes	Yes	Yes	Yes	Yes	Yes	Yes	Yes	Yes	Low
Livingstone et al 2016	1	1	1	-1	1	1	0	1	5	Yes	Yes	Yes	No	Yes	Yes	Yes	Yes	Yes	Yes	Low
Lv et al 2015	1	1	1	1	1	1	0	1	7	Yes	Yes	Yes	Yes	Yes	Yes	Yes	Yes	Yes	Yes	Low
Ma et al 2014	1	1	1	1	1	1	1	1	8	Yes	Yes	Yes	Yes	Yes	Yes	Yes	Yes	Yes	Yes	Low
Mansego et al 2015	1	1	1	1	1	1	-1	1	6	Yes	Yes	Yes	Yes	Yes	Yes	Yes	Yes	Yes	Yes	Low
Marcos-pasero et al 2019	1	1	1	1	-1	1	-1	1	4	Yes	Yes	Yes	Yes	Yes	Yes	Yes	Yes	Yes	Yes	Low
Martinez et al 2003	1	1	1	1	1	1	-1	1	6	Yes	Yes	Yes	Yes	No	Yes	Yes	Yes	Yes	Yes	Low
Masip et al 2020	1	1	1	1	1	1	0	1	7	Yes	Yes	Yes	Yes	Yes	Yes	Yes	Yes	Yes	Yes	Low
Merritt et al 2018	1	1	1	1	-1	1	0	1	5	Yes	Yes	Yes	Yes	Yes	Yes	Yes	Yes	Yes	Yes	Low
Miyaki et al 2005	1	1	1	1	1	1	-1	1	6	Yes	Yes	Yes	Yes	No	Yes	Yes	Yes	Yes	Yes	Low
Mollahosseini et al 2020	1	1	1	1	1	1	-1	1	6	Yes	Yes	Yes	Yes	Yes	Yes	Yes	No	Yes	Yes	Low
Mook-Kanamori et al	1	1	1	-1	1	1	0	1	5	Yes	Yes	Yes	No	Yes	Yes	Yes	Yes	Yes	Yes	Low
Mousavizadeh et al 2020	1	1	-1	1	1	1	0	1	5	Yes	Yes	Yes	Yes	Yes	Yes	Yes	Yes	Yes	Yes	Low
Muhammad et al 2019	1	1	1	1	-1	1	-1	1	4	Yes	Yes	Yes	Yes	No	Yes	Yes	Yes	Yes	Yes	Low
Nakamura et al 2016	1	1	1	1	1	1	0	1	7	Yes	Yes	Yes	Yes	Yes	Yes	No	Yes	Yes	Yes	Neutral

Nasreddine et al 2019	1	1	1	1	1	1	-1	1	7	Yes	Yes	Yes	Yes	Yes	Yes	No	Yes	Yes	Yes	Low
Nettleton et al 2015	1	1	1	1	1	1	1	1	8	Yes	Yes	Yes	Yes	Yes	Yes	Yes	Yes	Yes	Yes	Low
Nieters et al 2002	1	1	-1	1	1	1	-1	1	4	Yes	Yes	Yes	Yes	Yes	Yes	Yes	Yes	Yes	Yes	Low
Olsen et al 2016	1	1	1	1	-1	1	0	1	5	Yes	Yes	Yes	Yes	Yes	Yes	Yes	Yes	Yes	Yes	Low
Park et al 2013	1	1	1	1	1	1	1	1	8	Yes	Yes	Yes	Yes	Yes	Yes	Yes	Yes	Yes	Yes	Low
Park et al 2016	1	1	1	1	1	1	1	1	8	Yes	Yes	Yes	Yes	Yes	Yes	Yes	Yes	Yes	Yes	Low
Riedel et al 2013	1	1	1	1	-1	1	0	1	5	Yes	Yes	Yes	Yes	Yes	Yes	Yes	Yes	Yes	Yes	Low
Robitaille et al 2003	1	1	1	1	-1	1	-1	1	4	Yes	Yes	Yes	No	Yes	Yes	Yes	Yes	Yes	Yes	Low
Robitaille et al 2006	1	1	1	1	1	-1	-1	1	4	Yes	Yes	No	No	Yes	Yes	No	No	Yes	Yes	Neutral
Robitaille et al 2007	1	1	-1	1	1	1	-1	1	4	Yes	Yes	Yes	No	Yes	Yes	No	Yes	Yes	Yes	Neutral
Rocha et al 2018	1	1	1	1	1	1	0	1	7	Yes	Yes	Yes	Yes	Yes	Yes	Yes	Yes	Yes	Yes	Low
Rohde et al 2017	1	1	1	1	1	1	1	1	8	Yes	Yes	Yes	Yes	Yes	Yes	Yes	Yes	Yes	Yes	Low
Rukh et al 2013	1	1	1	1	1	1	1	1	8	Yes	Yes	Yes	Yes	Yes	Yes	Yes	Yes	Yes	Yes	Low
Rukh et al 2017	1	1	1	1	1	1	0	1	7	Yes	Yes	Yes	Yes	Yes	Yes	Yes	Yes	Yes	Yes	Low
Sanchez-Moreno et al 2010	1	1	1	1	-1	1	0	1	5	Yes	Yes	Yes	Yes	No	Yes	No	Yes	Yes	Yes	Neutral
Seral Cortes 2020	1	1	1	-1	1	1	-1	1	4	Yes	Yes	Yes	Yes	Yes	Yes	Yes	Yes	Yes	Yes	Low
Smith et al 2008	1	1	-1	1	1	1	-1	1	4	Yes	Yes	Yes	No	Yes	Yes	Yes	Yes	Yes	Yes	Low
Smith, Tucker, Arnett, et al 2013	1	1	1	1	1	1	0	1	7	Yes	Yes	Yes	Yes	Yes	Yes	Yes	Yes	Yes	Yes	Low
Smith, Tucker, Lee, et al 2013	1	1	1	1	1	1	-1	1	6	Yes	Yes	Yes	Yes	Yes	Yes	Yes	Yes	Yes	Yes	Low
Sonestedt et al 2009	1	1	1	1	1	1	0	1	7	Yes	Yes	Yes	Yes	Yes	Yes	Yes	Yes	Yes	Yes	Low

Sonestedt et al 2011	1	1	1	1	1	1	1	1	8	Yes	Yes	Yes	Yes	Yes	Yes	Yes	Yes	Yes	Yes	Low
Song et al 2007	1	1	1	1	1	1	-1	1	6	Yes	Yes	Yes	Yes	No	Yes	Yes	Yes	Yes	Yes	Low
Ding et al 2018	1	1	1	1	1	1	1	1	8	Yes	Yes	Yes	Yes	Yes	Yes	Yes	Yes	Yes	Yes	Low
Sotos-Prieto et al 2019	1	1	1	1	1	1	1	1	8	Yes	Yes	Yes	Yes	Yes	Yes	Yes	Yes	Yes	Yes	Low
Tao et al 2019	1	1	1	1	1	1	0	1	8	Yes	Yes	Yes	Yes	Yes	Yes	Yes	Yes	Yes	Yes	Low
Vaughan et al 2015	1	1	1	1	-1	1	-1	1	4	Yes	Yes	Yes	Yes	Yes	Yes	Yes	Yes	Yes	Yes	Low
Vázquez-Moreno et al 2020	1	1	1	1	-1	1	-1	1	4	Yes	Yes	Yes	No	No	Yes	Yes	Yes	Yes	Yes	Low
Wang, Garcia-Bailo, et al 2014	1	1	1	1	-1	1	0	1	5	Yes	Yes	Yes	Yes	Yes	Yes	Yes	Yes	Yes	Yes	Low
Wang, Tang, et al 2014	1	1	1	1	1	1	-1	1	6	Yes	Yes	Yes	Yes	No	Yes	Yes	Yes	Yes	Yes	Low
Wang et al 2015	1	1	1	1	1	1	0	1	7	Yes	Yes	Yes	Yes	Yes	Yes	Yes	Yes	Yes	Yes	Low
Wang et al 2017	1	1	1	-1	-1	1	1	1	4	Yes	Yes	Yes	Yes	Yes	Yes	Yes	Yes	Yes	Yes	Low
Wang et al 2018	1	1	1	-1	-1	1	1	1	4	Yes	Yes	Yes	Yes	Yes	Yes	Yes	Yes	Yes	Yes	Low
Warodomwichit et al 2009	1	1	1	-1	1	1	0	1	5	Yes	Yes	Yes	Yes	Yes	Yes	Yes	Yes	Yes	Yes	Low
Wu et al 2017	1	1	1	1	-1	1	1	1	6	Yes	Yes	Yes	Yes	Yes	Yes	Yes	Yes	Yes	Yes	Low
Yarizadeh et al 2021	1	1	1	1	-1	1	-1	1	4	Yes	Yes	Yes	Yes	Yes	Yes	Yes	No	Yes	Yes	Low
Young et al 2016	1	1	1	1	1	1	1	1	8	Yes	Yes	Yes	Yes	Yes	Yes	Yes	Yes	Yes	Yes	Low
Zhang et al 2015	1	1	1	1	-1	1	0	1	5	Yes	Yes	Yes	Yes	Yes	Yes	Yes	Yes	Yes	Yes	Low
Zhu, Xue, Guo, Deng, et al 2020	1	1	1	1	1	1	0	1	7	Yes	Yes	Yes	Yes	Yes	Yes	Yes	Yes	Yes	Yes	Low
Zhu, Xue, Guo, and	1	1	1	1	1	1	-1	1	6	Yes	Yes	Yes	Yes	No	Yes	Yes	Yes	Yes	Yes	Low

Yang 2020																				
**Interventional trials (n=60)**																				
Abete et al 2009	1	1	1	1	1	1	0	1	7	Yes	Yes	No	No	No	Yes	No	No	Yes	Yes	Neutral
Arias et al 2017	1	1	1	1	1	1	-1	1	6	Yes	Yes	No	No	No	Yes	Yes	Yes	Yes	Yes	Neutral
Cha et al 2006	1	1	-1	1	1	1	0	1	5	Yes	Yes	No	No	No	Yes	Yes	No	Yes	Yes	Neutral
Cha et al 2007	1	1	1	1	1	1	1	1	8	Yes	Yes	No	Yes	No	Yes	Yes	No	Yes	Yes	Neutral
Cha et al 2014	1	1	1	1	-1	1	-1	1	4	Yes	Yes	Yes	Yes	Yes	Yes	Yes	Yes	Yes	Yes	Low
De Luis, Aller, Izaola, Sagrado, et al 2012	1	1	1	1	1	-1	0	1	5	Yes	Yes	No	Yes	No	Yes	Yes	Yes	Yes	Yes	Neutral
De Luis, Aller, Izaola, de la Fuente, et al 2012	1	1	1	1	1	-1	1	1	6	Yes	Yes	No	Yes	No	Yes	Yes	Yes	Yes	Yes	Neutral
De Luis, Aller, Izaola, Conde, et al 2013	1	1	1	1	1	-1	0	1	5	Yes	Yes	No	Yes	No	Yes	Yes	Yes	Yes	Yes	Neutral
De Luis, Aller, Izaola, Sagrado, et al 2013	1	1	1	1	1	-1	0	1	5	Yes	Yes	No	Yes	No	Yes	Yes	Yes	Yes	Yes	Neutral
De Luis, Izaola, et al 2013	1	1	1	1	1	-1	0	1	5	Yes	Yes	No	Yes	No	Yes	Yes	Yes	Yes	Yes	Neutral
De Luis et al 2014	1	1	1	1	1	-1	0	1	5	Yes	Yes	No	Yes	No	Yes	Yes	Yes	Yes	Yes	Neutral
De Luis et al 2015	1	1	1	1	1	-1	0	1	5	Yes	Yes	No	Yes	No	Yes	Yes	Yes	Yes	Yes	Neutral
De Luis et al 2016a	1	1	1	1	1	-1	0	1	5	Yes	Yes	No	Yes	No	Yes	Yes	Yes	Yes	Yes	Neutral
De Luis et al 2016b	1	1	1	1	1	-1	0	1	5	Yes	Yes	No	Yes	No	Yes	Yes	Yes	Yes	Yes	Neutral

De Luis, Izaola, et al 2018	1	1	1	1	1	-1	0	1	5	Yes	Yes	No	Yes	No	Yes	Yes	Yes	Yes	Yes	Neut ral
De Luis, Fernánde z Ovalle, et al. 2018	1	1	1	1	1	-1	0	1	5	Yes	Yes	No	Yes	No	Yes	Yes	Yes	Yes	Yes	Neut ral
De Luis et al 2019	1	1	1	1	1	-1	0	1	5	Yes	Yes	No	Yes	No	Yes	Yes	Yes	Yes	Yes	Neut ral
Di Renzo et al 2013	1	1	1	1	-1	-1	-1	1	2	Yes	Yes	No	Yes	No	Yes	Yes	No	Yes	Yes	Neut ral
Di Renzo et al 2018	1	1	1	0	1	1	0	1	6	Yes	Yes	Yes	Yes	No	Yes	Yes	Yes	Yes	Yes	Low
Frankwic h et al 2016	1	1	0	0	-1	1	-1	1	2	Yes	Yes	Yes	Yes	No	Yes	Yes	Yes	Yes	Yes	Low
Goni et al 2018	1	1	1	1	1	1	0	1	7	Yes	Yes	Yes	No	No	Yes	Yes	No	Yes	Yes	Low
Goni et al 2019	1	1	1	1	1	1	1	1	8	Yes	Yes	Yes	Yes	Yes	Yes	Yes	No	Yes	Yes	Low
Grau et al 2009	1	1	1	0	1	1	0	1	6	Yes	Yes	Yes	Yes	No	Yes	Yes	Yes	Yes	Yes	Low
Grau et al 2010	1	1	1	1	1	1	1	1	8	Yes	Yes	Yes	Yes	No	Yes	Yes	Yes	Yes	Yes	Low
Guinotte et al 2003	1	1	1	1	-1	1	-1	1	4	Yes	Yes	No	Yes	No	Yes	Yes	Yes	Yes	Yes	Neut ral
Hamada et al 2011	1	1	1	1	1	-1	-1	1	4	Yes	Yes	No	No	No	Yes	Yes	No	Yes	Yes	Neut ral
Heianza et al 2016	1	1	1	1	1	1	1	1	8	Yes	Yes	Yes	Yes	Yes	Yes	Yes	Yes	Yes	Yes	Low
Heianza et al 2017	1	1	1	1	1	1	1	1	8	Yes	Yes	Yes	Yes	Yes	Yes	Yes	Yes	Yes	Yes	Low
Hernande x-Guerrero et al 2018	1	1	-1	0	-1	0	0	1	1	Yes	Yes	No	Yes	No	Yes	Yes	Yes	Yes	Yes	Neut ral
Huang et al 2018	1	1	1	-1	1	1	1	1	6	Yes	Yes	Yes	Yes	Yes	Yes	Yes	No	Yes	Yes	Low
Labayen et al 2015	1	0	-1	-1	1	1	0	1	2	Yes	Yes	No	Yes	No	Yes	Yes	No	Yes	Yes	Neut ral
Lee et al 2012	1	1	1	1	-1	1	0	1	5	Yes	Yes	Yes	Yes	Yes	Yes	Yes	Yes	Yes	Yes	Low
Li et al 2020	1	1	1	0	1	1	1	1	7	Yes	Yes	Yes	Yes	Yes	Yes	Yes	Yes	Yes	Yes	Low
Lin et al 2015	1	1	1	1	1	1	1	1	8	Yes	Yes	Yes	No	Yes	Yes	Yes	Yes	Yes	Yes	Low

Lisboa et al 2020	1	1	0	0	-1	1	-1	1	2	Yes	Yes	Yes	Yes	Yes	Yes	Yes	Yes	Yes	Yes	Low
Mammes et al 2001	1	1	-1	1	1	-1	0	1	3	Yes	Yes	No	Yes	No	Yes	Yes	Yes	Yes	Yes	Neutral
Martinez-Lopez et al 2013	1	1	1	1	1	1	0	1	7	Yes	Yes	No	No	No	Yes	Yes	No	Yes	Yes	Neutral
Matsuo et al 2009	1	1	1	1	1	1	0	1	7	Yes	Yes	No	No	No	Yes	Yes	Yes	Yes	Yes	Neutral
Mattei et al 2012	1	1	1	1	1	1	1	1	8	Yes	Yes	Yes	Yes	Yes	Yes	Yes	Yes	Yes	Yes	Low
Namazi et al 2017	1	1	1	0	-1	1	-1	1	3	Yes	Yes	Yes	Yes	Yes	Yes	Yes	Yes	Yes	Yes	Low
Nikpay et al 2020	1	1	1	1	1	1	1	1	8	Yes	Yes	No	Yes	No	Yes	Yes	No	Yes	Yes	Neutral
Ramos-Lopez et al 2019	1	1	1	1	-1	1	0	1	5	Yes	Yes	Yes	Yes	No	Yes	Yes	Yes	Yes	Yes	Low
Rauhio et al 2013	1	1	-1	1	-1	1	0	1	3	Yes	Yes	Unclear	Yes	Unclear	Yes	Yes	Yes	Yes	Yes	Low
Razquin et al 2010	1	1	1	1	1	1	1	1	8	Yes	Yes	Yes	Yes	Yes	Yes	Yes	Yes	Yes	Yes	Low
Rodrigues et al 2018	1	1	1	1	1	1	0	1	7	Yes	Yes	Yes	Yes	Yes	Yes	Yes	Yes	Yes	Yes	Low
Ruiz et al 2011	1	1	-1	1	1	-1	0	1	3	Yes	Yes	No	Yes	No	Yes	Yes	Yes	Yes	Yes	Neutral
San-Cristobal et al 2017	1	1	1	1	1	1	1	1	8	Yes	Yes	Yes	Yes	No	Yes	Yes	Yes	Yes	Yes	Low
Seip et al 2008	1	1	1	-1	1	1	0	1	5	Yes	Yes	Yes	No	No	Yes	Yes	No	Yes	Yes	Low
Soenen et al 2009	1	1	-1	1	1	1	0	1	5	Yes	Yes	No	Unclear	No	Yes	Yes	Yes	Yes	Yes	Neutral
Solis et al 2008	1	1	1	1	-1	1	-1	1	4	Yes	Yes	No	Yes	No	Yes	Yes	Yes	Yes	Yes	Neutral
Stocks et al 2012	1	1	1	1	1	1	1	1	8	Yes	Yes	Yes	Yes	No	Yes	Yes	Yes	Yes	Yes	Low
Svendstrup et al 2018	1	1	1	1	1	1	1	1	8	Yes	Yes	Yes	Yes	No	Yes	Yes	Yes	Yes	Yes	Low
Tan et al 2020	1	1	1	0	1	1	0	1	6	Yes	Yes	Yes	Yes	No	Yes	Yes	Yes	Yes	Yes	Low
Teixeira et al 2020	1	1	1	1	1	1	0	1	7	Yes	Yes	No	Yes	No	Yes	Yes	No	Yes	Yes	Neutral
Teixeira et al 2020	1	1	1	1	1	1	0	1	7	Yes	Yes	No	Yes	No	Yes	Yes	No	Yes	Yes	Neutral

Tsuzaki et al 2009	1	1	-1	1	1	-1	-1	1	2	Yes	Yes	No	No	No	Yes	Yes	No	Yes	Yes	Neutral
Verhoef et al 2014	1	1	1	1	1	0	0	1	6	Yes	Yes	No	Yes	No	Yes	Yes	Yes	Yes	Yes	Neutral
Xinli et al 2001	1	1	1	1	1	1	0	1	7	Yes	Yes	No	Yes	No	Yes	Yes	Yes	Yes	Yes	Neutral
Yoon et al 2007	1	1	-1	1	-1	1	1	1	4	Yes	Yes	No	No	No	Yes	Yes	No	Yes	Yes	Neutral
Zhang et al 2012	1	1	1	1	1	1	1	1	8	Yes	Yes	Yes	No	Yes	Yes	Yes	Yes	Yes	Yes	Low
